# Data on the effect of high-pressure torsion processing on secondary cast Al–10%Si– Cu piston alloy: Methods, microstructure and mechanical characterizations

**DOI:** 10.1016/j.dib.2019.104160

**Published:** 2019-07-03

**Authors:** F.M. Mwema, T.O. Mbuya, E.T. Akinlabi, P.A.S. Reed, J.O. Obiko

**Affiliations:** a Department of Mechanical Engineering Science, University of Johannesburg, South Africa; b Department of Mechanical Engineering, Dedan Kimathi University of Technology, Nyeri, Kenya; c Department of Mechanical and Manufacturing Engineering, University of Nairobi, Kenya; d Mechanical Engineering, Engineering Group, Faculty of Engineering and Environment, University of Southampton, UK; e Department of Mining, Materials and Petroleum Engineering, Jomo Kenyatta University of Agriculture and Technology, Nairobi, Kenya

**Keywords:** Aluminum alloys, High-pressure torsion (HPT), Severe plastic deformation (SPD), Grain refinement, Microhardness

## Abstract

The dataset presented here shows the microstructure and mechanical properties of secondary (recycled) cast aluminum-silicon (Al–Si) piston alloys processed through severe plastic deformation technique, known as high-pressure torsion (HPT). The HPT processing was undertaken for 1/4, 1/2, 1 and 10 turns of the lower anvil (rotating at constant speed of 1rpm) while the upper anvil maintained at a normal pressure of 3.0 GPa. The data on microstructural evolution obtained at the central region and edge of the circular (disk) HPT sample were obtained using optical and scanning electron microscopy and these data are presented here. The data on the analysis of the particle shape, sizes and distribution from the micrographs using ImageJ software are also presented. Data on mechanical properties characterized using Vickers microhardness measurement across the surface of HPT sample are also shown. Pictures depicting the microhardness measurement scheme, high-pressure torsion facility and sample nomenclature are presented.

**Table:** Specifications table

Subject area	*Mechanical Engineering*
More specific subject area	*Mechanical metallurgy*
Type of data	*Image, graph, figure, picture*
How data was acquired	*Optical microscope (Olympus BX5), SEM/EDS (JEOL JSM 6500F microscope at an accelerating voltage of* 15 kV*), Microhardness (Matsuzawa Seiki Co. Ltd, Vickers indenter), ImageJ (open source image analyses software)*
Data format	*Raw, filtered, analyzed,*
Experimental factors	*For high-pressure processing, microscopy and microhardness measurements, the samples were ground to SiC grade #1200, washed in acetone, polished to 1/4* m *diamond paste and oxide polish suspension finish (0.05* m*) and sonicated in ultra-sound bath in distilled water and dried.*
Experimental features	*High-pressure torsion was undertaken for 1/4, 1/2 1 and 10 turns on a recycled Al–Si piston alloy with the following composition: 10.6%Si, 1.36%Cu, 1.08%Ni, 0.78%Mg, 1.06%Fe, 0.08%Mn,* *0.03%Cr, 0.06%Ti, 0.02%Sn, 0.09%Zn and 0.04%K. This is a typical piston alloy according to AE413 standards.*
Data source location	*University of Southampton, UK and Department of Mechanical and Manufacturing Engineering, University of Nairobi, Kenya*
Data accessibility	*Data is with this article*

**Table:** 

**Value of the data** • The data can be used as a basis for understanding the evolution of microstructure and mechanical properties of recycled (secondary) cast Al–10%Si–Cu piston alloys processed through high-pressure torsion (HPT) at different number of turns. • The data can be used as a reference study for improvement of behavior of recycled aluminum alloys through HPT and evolution of homogeneity across the surface of the processed samples. • The data will be beneficial to researchers, scientists and industrialists interested in understanding, interpreting and utilizing high pressure torsion in processing of recycled aluminum alloys. • The new method of evaluation of microhardness and the corresponding hardness data presented here will be beneficial to researchers to analyze the homogeneity evolution with the strains. This will further enhance development of HPT processes for effective and uniform straining of alloys for industrial applications.

## 1 Data

The data presented here is on the evolution of the properties of secondary aluminum-silicon.

**Fig. 1:**
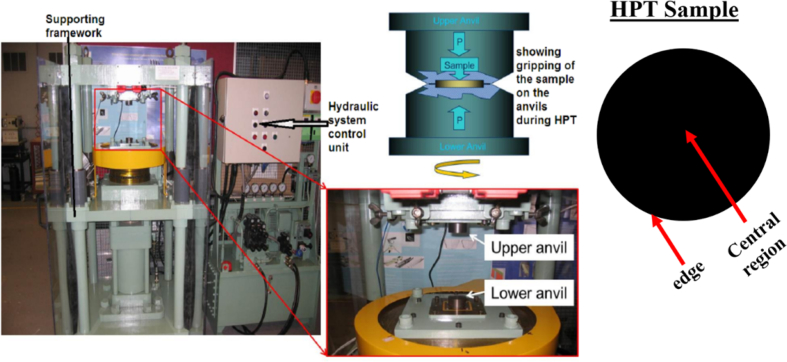
High-pressure torsion facility at the University of Southampton used in this work. The microhardness and microstructure measurements were undertaken on the central region and edge of the sample as shown.

**Fig. 2:**
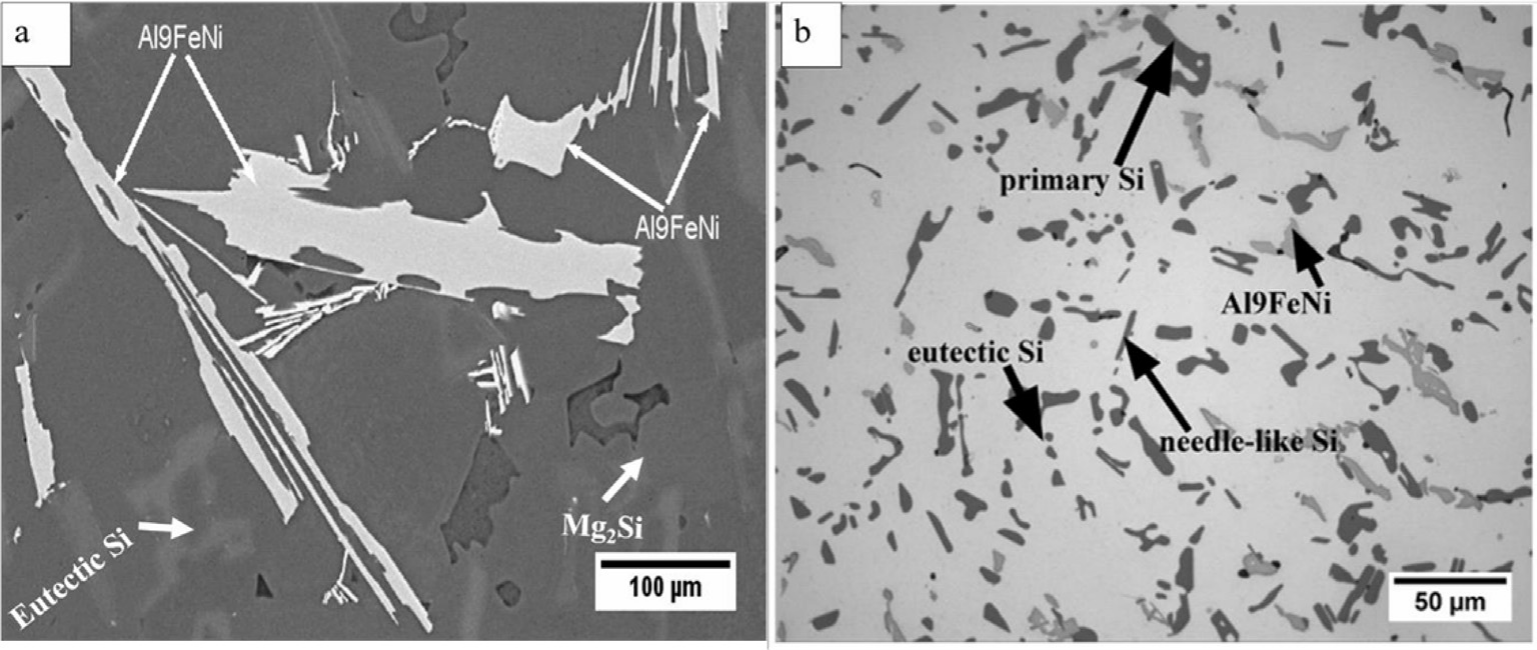
(a) SEM micrograph showing various intermetallic phases present in the unprocessed/as-received Al–Si alloy. The phases were identified through EDS mounted on the SEM facility (b) Optical micrograph showing different morphologies of the silicon particles in the as received alloy.

**Fig. 3:**
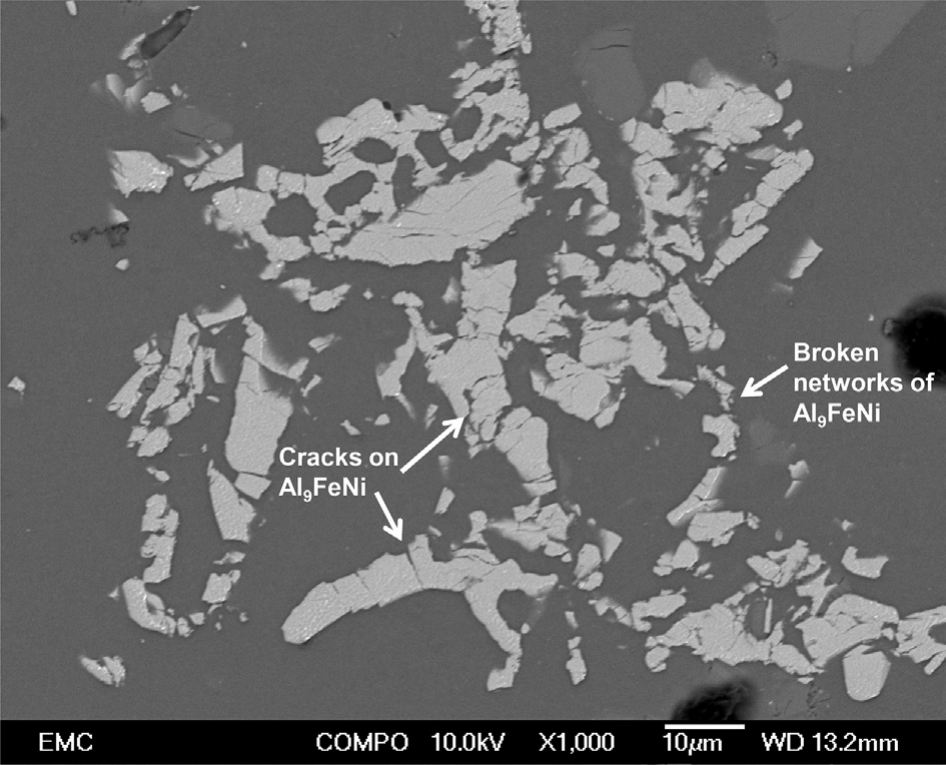
SEM micrograph at the edge of the recycled (secondary) Al–10%Si–Cu piston alloy after 1/4 HPT turn. The breakdown of various phases is illustrated.

**Fig. 4:**
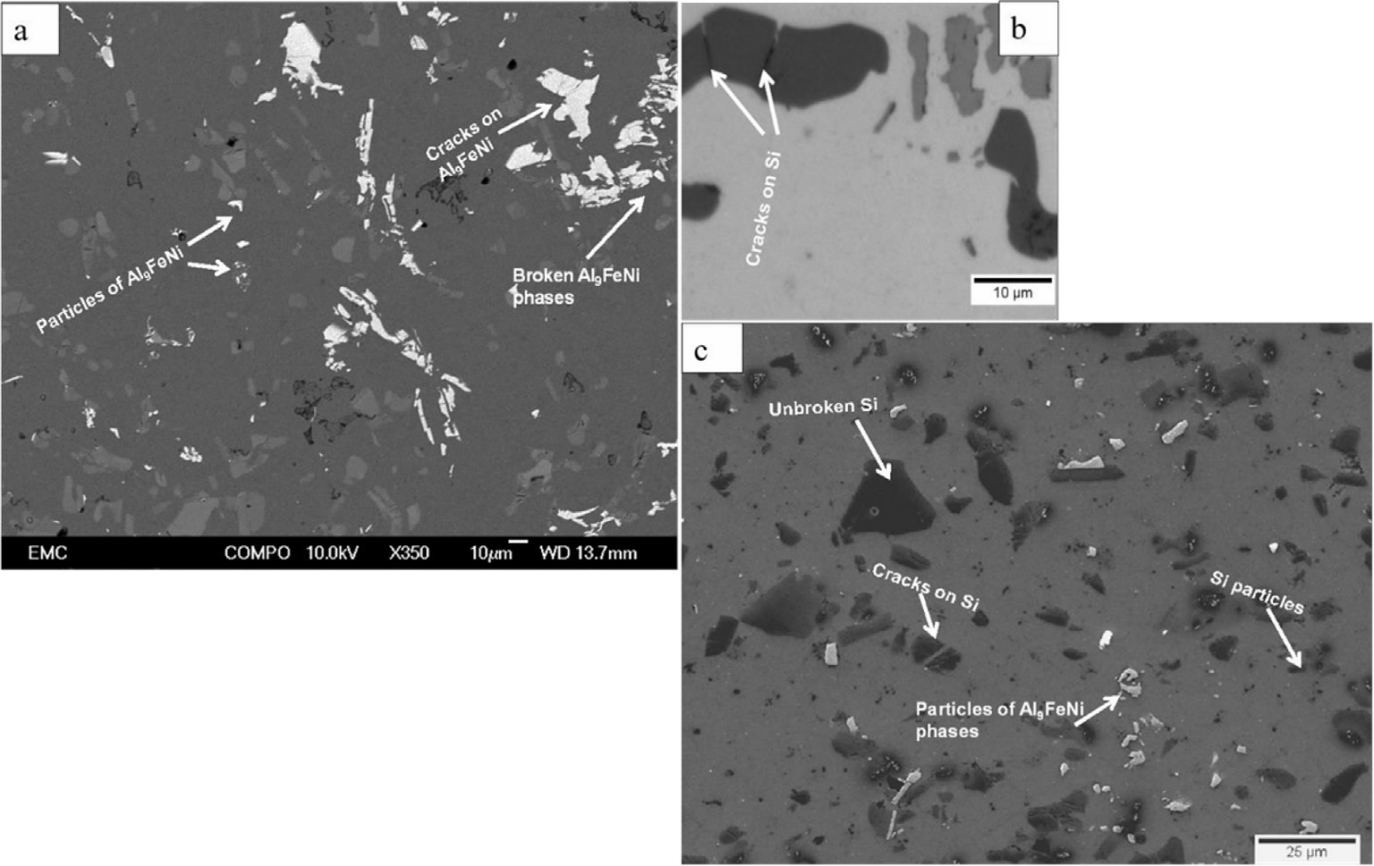
(a) SEM micrograph showing the microstructural transformations at the central region of the specimen after 1/2 turn (b) Exploded optical micrograph illustrating cracks on the silicon particles at the central region of the HPT specimen after 1/2 turn (c) SEM micrograph showing the microstructural features at the edge of the HPT specimen after 1/2 turn.

**Fig. 5:**
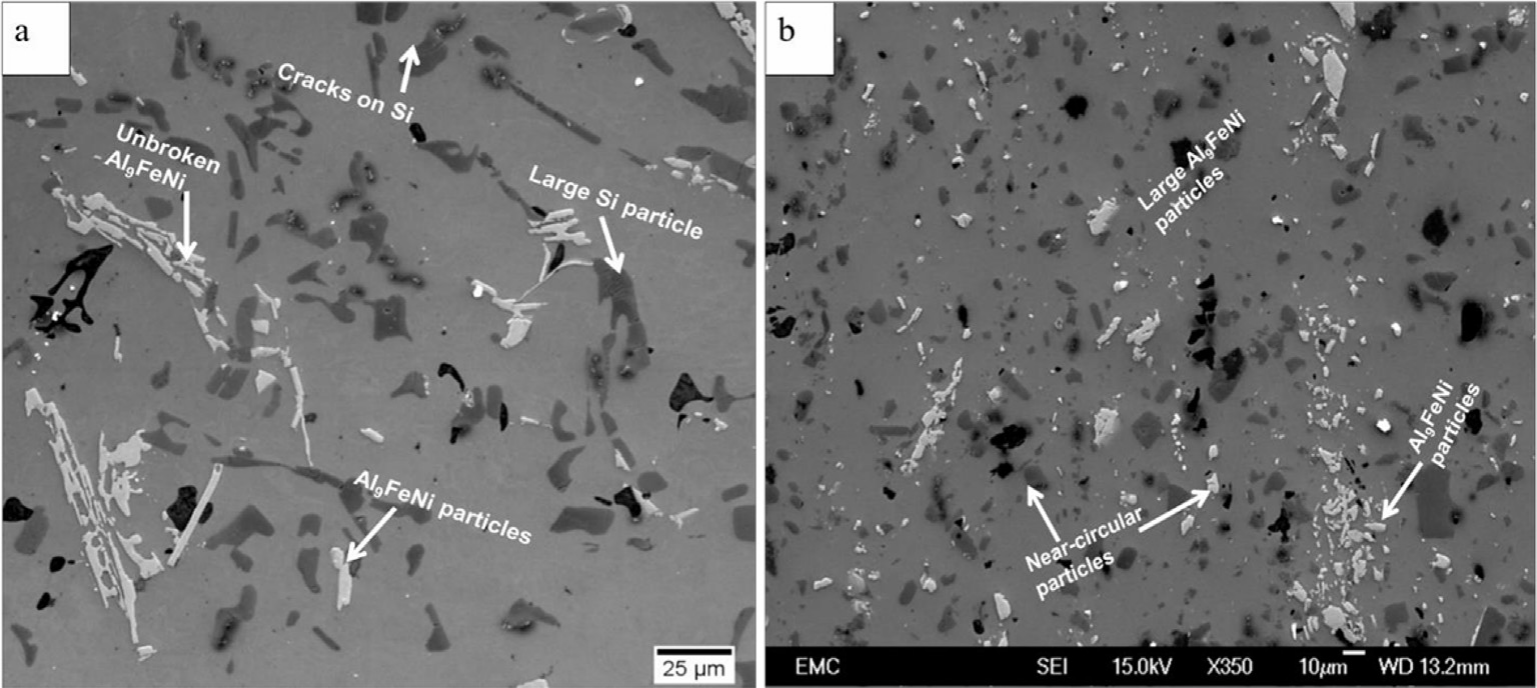
SEM micrographs showing breakdown of intermetallic phases at the (a) central region and (b) edge of the HPT specimen after 1 turn.

**Fig. 6:**
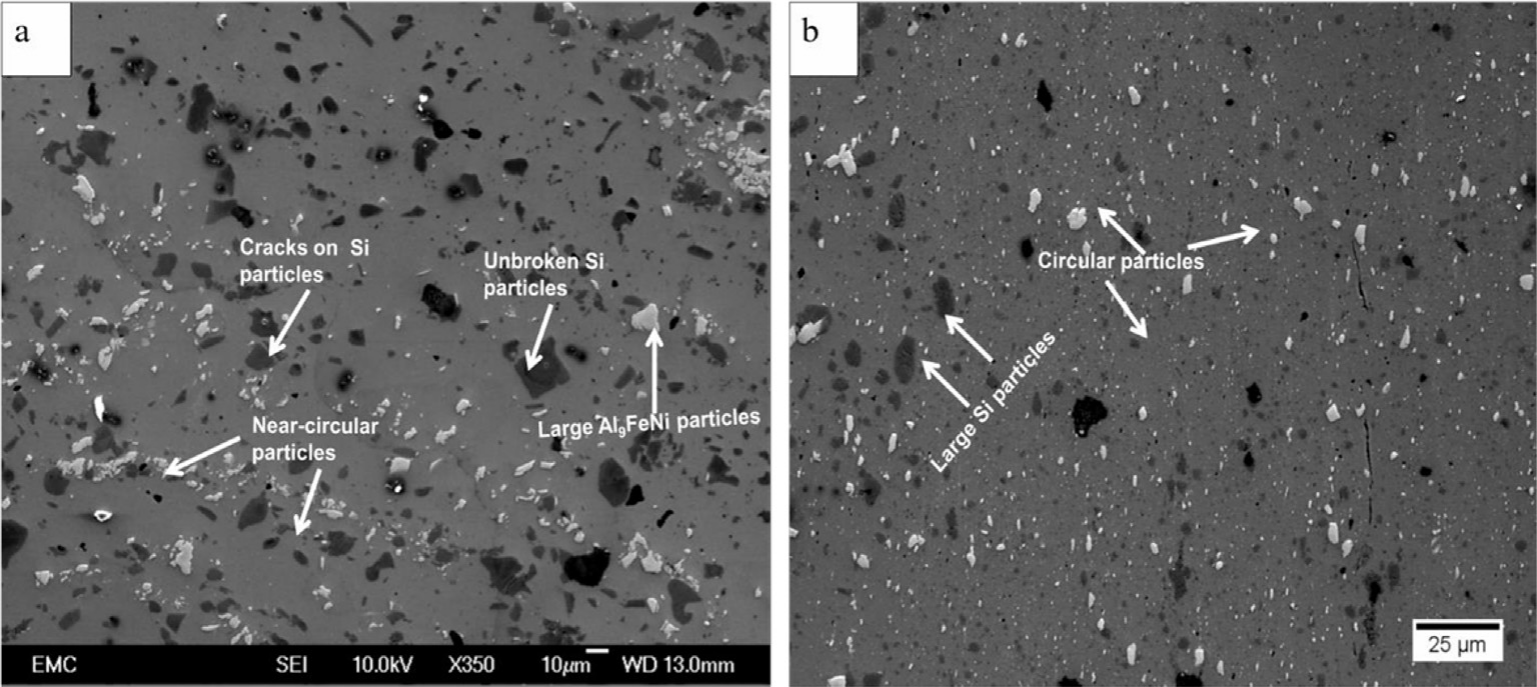
(a) SEM micrograph showing microstructural transformation at the central region of the HPT specimen after 10 turns. The microstructure is considerably refined and contains near-circular intermetallic and silicon particles (b) SEM micrograph showing a refined microstructure at the edge of the HPT specimen after 10 turns. The microstructure is characterized by circular and small intermetallic particles; however, there are occasional and scattered larger and unbroken Si-rich phases and particles.

**Fig. 7:**
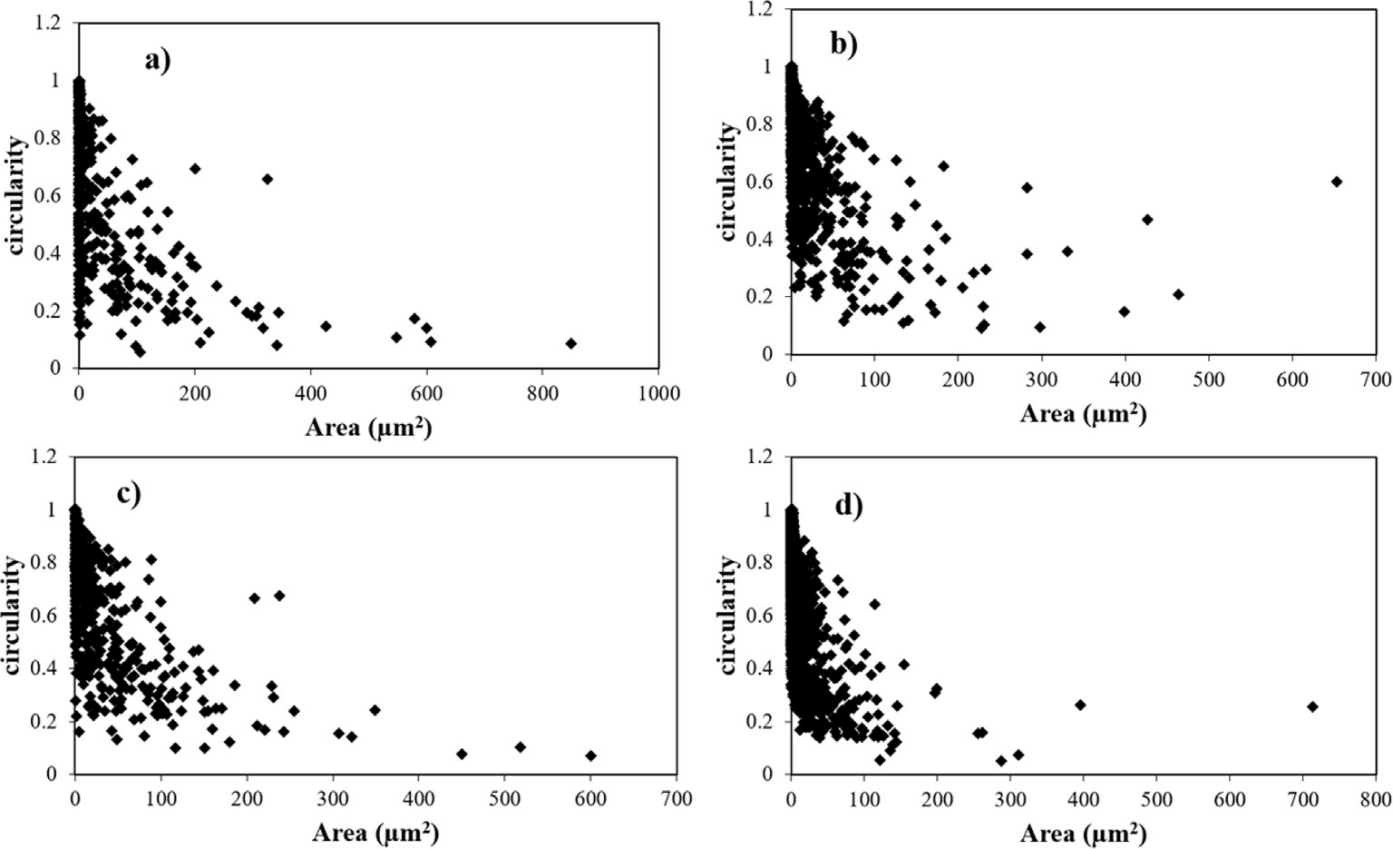
Relationship between circularity and particle area at the central region for (a) 1/4 turn HPT specimen and (b) 10 turn HPT specimen, at the edge for (c) 1/4 turn HPT specimen and (d) 10 turn HPT specimen.

**Fig. 10:**
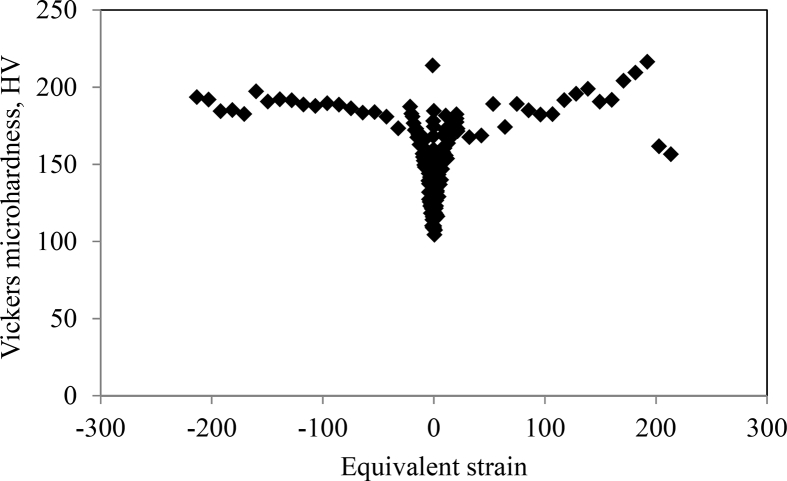
Vickers microhardness variations with the equivalent strain for the recycled Al–10%Si–Cu. The zero mark represents the strain at center of the specimen. The negative and positive sign values indicate equivalent strains on opposite but equal locations along the specimen diameter relative to the center.

Piston alloy severely deformed through high-pressure torsion (HPT). The picture of the HPT facility used is shown in Fig. 1. Fig. 2 represent the optical and SEM micrographs of the unprocessed secondary Al–Si piston alloy showing the dominant phases identified via EDS technique. The SEM image showing the breakdown of phases at the edge of the alloy after 1/4 turn is represented in Fig. 3. Fig. 4 shows the various microstructural transformations at the central and periphery of the samples after 1/2 turn. Fig. 5, Fig. 6 represent the microstructure changes at the center and edge of the samples processed at 1 and 10 turns respectively. The data of circularity analyses determined through the ImageJ software for the microstructure of the samples at different number of turns of the HPT process are presented in Table S1 and plotted in Fig. 7. Microhardness data and corresponding profiles along the radial surface are shown in Table 1 and Fig. 9. The dataset on variation of the hardness with equivalent strains are also shown in Table 2 and Fig. 10.

**Table 1:** Microhardness data and their corresponding error bars for unprocessed samples and samples processed at 1/4, 1/2, 1 and 10 high-pressure turns (Plots in Fig. 9).

r(mm)	Without HPT processing				¼ turn				½ turn				1 turn				10 turns			
	LH (HV)	Av.HV (HV)	SD	95% C.F	LH (HV)	Av.HV (HV)	SD	95% C.F	LH (HV)	Av.HV (HV)	SD	95% C.F	LH (HV)	Av.HV (HV)	SD	95% C.F	LH (HV)	Av.HV (HV)	SD	95% C.F
−5	91.54				161.69				139.69				161.14				251.25			
	96.17				157.11				168.52				184.87				209.30			
	103.94				160.60				171.49				182.87				201.03			
	95.04	96.67	9.83	15.64	167.64	161.76	12.72	20.24	193.24	168.24	12.97	23.83	183.87	178.19	13.35	21.24	194.69	214.07	14.63	26.88
−4.75	96.17				158.97				167.93				181.88				170.00			
	95.54				157.11				139.69				184.87				209.30			
	86.37				154.50				156.06				194.32				180.57			
	95.29	93.34	9.66	15.37	155.80	156.59	12.51	19.91	174.85	159.63	12.63	23.21	188.64	187.43	13.69	25.15	178.63	184.63	13.59	24.96
−4.5	95.54				155.80				159.51				183.53				200.65			
	113.67				158.97				167.93				181.88				170.00			
	88.00				158.44				155.54				183.87				208.49			
	102.96	100.04	10.00	15.91	157.11	157.58	12.55	19.97	164.20	161.80	12.72	23.37	181.88	182.79	13.52	24.84	195.05	193.55	13.91	25.56
−4.25	113.67				149.72				149.48				176.41				191.09			
	98.09				155.80				155.54				181.88				200.65			
	98.22				146.34				163.92				183.87				194.32			
	102.96	103.24	10.16	16.17	153.48	151.33	12.30	19.57	158.44	156.84	12.52	23.01	181.88	181.01	13.45	24.72	181.88	191.99	13.86	25.46
−4	98.09				140.81				152.21				165.62				172.70			
	88.67				149.72				151.96				177.36				191.09			
	91.19				156.84				158.44				174.54				188.64			
	95.92	93.47	9.67	15.38	152.21	149.89	12.24	19.48	156.58	154.80	12.44	22.86	188.99	176.63	13.29	24.42	185.55	184.50	13.58	24.95
−3.75	88.67				144.69				150.71				169.70				185.21			
	88.67				140.81				152.21				165.62				172.70			
	88.00				137.72				151.21				174.23				192.52			
	108.80	93.54	9.67	15.39	152.97	144.05	12.00	19.10	155.28	152.35	12.34	22.68	178.95	172.13	13.12	24.10	190.39	185.21	13.61	25.00
−3.5	88.67				135.16				151.46				176.41				183.20			
	100.21				144.69				151.46				169.70				185.21			
	101.02				135.58				145.87				176.73				183.20			
	98.09	97.00	9.85	15.67	134.11	137.38	11.72	18.65	149.48	149.56	12.23	22.47	169.40	173.06	13.16	24.17	178.95	182.64	13.51	24.83
−3.25	100.21				137.72				145.16				160.87				195.42			
	101.30				136.22				151.46				176.41				183.20			
	94.18				140.81				145.63				163.64				197.63			
	91.54	96.81	9.84	15.65	145.16	139.98	11.83	18.82	150.71	148.24	12.18	22.37	169.11	167.51	12.94	23.78	213.00	197.31	14.05	25.81
−3	101.30				130.64				151.71				164.77				193.96			
	90.14				137.72				145.16				160.87				195.42			
	92.01				127.11				154.24				177.36				185.89			
	97.19	95.16	9.75	15.52	133.90	132.34	11.50	18.30	144.46	148.89	12.20	22.42	173.62	169.15	13.01	23.89	187.60	190.72	13.81	25.37
−2.75	90.03				152.97				155.28				162.80				192.52			
	86.91				131.64				151.71				164.77				193.96			
	91.19				144.69				149.48				165.34				192.88			
	91.65	89.94	9.48	15.09	137.50	141.70	11.90	18.94	148.02	151.12	12.29	22.58	158.44	162.84	12.76	23.44	189.34	192.17	13.86	25.47
−2.5	88.45				130.44				148.74				179.28				177.99			
	92.48				146.58				155.28				162.80				192.52			
	95.54				135.58				154.50				167.06				188.99			
	103.38	94.96	9.74	15.50	138.16	137.69	11.73	18.67	155.02	153.38	12.38	22.75	167.06	169.05	13.00	23.89	206.49	191.50	13.84	25.42
−2.25	92.48				132.25				156.84				157.64				186.57			
	89.01				130.44				148.74				179.28				177.99			
	93.69				133.28				142.84				161.97				199.13			
	91.07	91.56	9.57	15.22	132.25	132.06	11.49	18.28	149.97	149.60	12.23	22.47	172.40	167.82	12.95	23.80	191.45	188.79	13.74	25.24
−2	89.01				124.83				143.53				164.20				189.69			
	91.30				132.25				156.84				157.64				186.57			
	93.81				132.05				145.63				168.23				192.16			
	94.79	92.23	9.60	15.28	135.79	131.23	11.46	18.23	148.99	148.75	12.20	22.41	175.79	166.46	12.90	23.70	183.20	187.90	13.71	25.18
−1.75	91.30				128.66				138.37				164.77				183.87			
	100.62				124.83				143.53				164.20				189.69			
	89.80				121.71				141.71				167.06				185.21			
	93.32	93.76	9.68	15.41	121.53	124.18	11.14	17.73	133.90	139.38	11.81	21.69	167.35	165.85	12.88	23.66	199.51	189.57	13.77	25.29
−1.5	100.62				118.88				125.58				155.80				191.09			
	92.48				128.66				138.37				164.77				183.87			
	105.96				118.88				123.53				157.64				183.20			
	95.92	98.74	9.94	15.81	122.07	122.12	11.05	17.58	121.53	127.26	11.28	20.72	159.51	159.43	12.63	23.20	196.52	188.67	13.74	25.23
−1.25	92.48				125.40				122.80				149.72				193.24			
	89.57				118.88				125.58				155.80				191.09			
	91.65				119.58				123.35				147.78				179.60			
	88.67	90.60	9.52	15.14	122.07	121.48	11.02	17.54	131.04	125.69	11.21	20.60	148.26	150.39	12.26	22.53	181.22	186.29	13.65	25.07
−1	89.57				116.32				126.72				136.86				174.85			
	90.72				125.40				125.21				149.72				193.24			
	89.92				118.19				116.15				148.26				183.53			
	93.57	90.94	9.54	15.17	124.09	121.00	11.00	17.50	123.90	123.00	11.09	20.37	149.97	146.20	12.09	22.21	182.54	183.54	13.55	24.89
−0.75	90.72				117.68				105.82				134.11				179.28			
	98.22				116.32				126.72				136.86				174.85			
	85.94				115.15				116.82				123.72				189.69			
	91.54	91.60	9.57	15.23	107.29	114.11	10.68	17.00	123.90	118.32	10.88	19.98	133.07	131.94	11.49	18.28	191.45	183.82	13.56	24.91
−0.5	98.22				115.81				117.16				119.93				179.92			
	87.78				117.85				140.14				134.11				179.28			
	94.92				116.49				133.69				122.44				186.23			
	95.66	94.15	9.70	15.44	114.16	116.08	10.77	17.14	107.74	124.68	11.17	20.51	133.69	127.54	11.29	20.75	178.31	180.94	13.45	24.71
−0.25	87.78				108.95				106.40				120.64				175.47			
	99.41				115.81				117.16				119.93				179.92			
	93.81				112.70				103.66				124.65				167.64			
	85.41	91.60	9.57	15.23	99.14	109.15	10.45	16.62	113.35	110.14	10.49	19.28	126.53	122.94	11.09	20.37	170.29	173.33	13.17	24.19
0	99.41				106.70				95.54				120.46				164.20			
	84.25				108.95				106.40				120.64				175.47			
	96.42				115.98				124.83				114.49				171.79			
	93.20	93.32	9.66	15.37	102.12	108.44	10.41	16.57	108.95	108.93	10.44	19.17	125.96	120.39	10.97	20.16	180.90	173.09	13.16	24.17
0.25	84.25				111.91				104.09				124.09				177.04			
	91.07				119.58				95.54				120.46				164.20			
	92.60				94.18				107.44				113.19				186.91			
	95.66	90.90	9.53	15.17	114.49	110.04	10.49	16.69	110.34	104.35	10.22	18.77	118.88	117.51	10.84	19.91	187.95	179.69	13.40	24.63
0.5	91.07				112.22				108.65				124.09				161.69			
	83.94				111.91				104.09				120.46				177.04			
	92.84				122.98				111.91				113.19				182.21			
	99.68	91.88	9.59	15.25	109.87	114.25	10.69	17.01	105.09	107.43	10.36	19.04	118.88	117.51	10.84	19.91	164.20	174.49	13.21	24.27
0.75	83.94				123.17				122.26				117.33				173.01			
	85.94				112.22				108.65				117.85				161.69			
	88.34				113.67				121.89				111.27				164.20			
	89.80	87.00	9.33	14.84	112.86	115.48	10.75	17.10	133.07	121.47	11.02	20.25	119.76	116.29	10.78	19.81	176.73	167.54	12.94	23.78
1	85.94				113.67				124.27				137.50				170.00			
	103.66				123.17				122.26				120.64				173.01			
	93.32				118.71				134.53				129.05				169.70			
	89.69	93.15	9.65	15.36	119.23	118.69	10.89	17.33	125.77	126.71	11.26	20.68	137.50	129.07	11.36	20.87	171.79	171.50	13.10	24.06
1.25	103.66				123.90				140.36				138.16				170.00			
	95.29				113.67				124.09				137.50				170.00			
	98.22				145.63				129.64				130.44				163.64			
	115.15	103.08	10.15	16.15	131.85	128.76	11.35	18.05	134.53	132.15	11.50	21.12	142.84	136.93	11.70	21.50	169.11	167.58	12.95	23.78
1.5	95.29				125.40				134.95				143.07				178.63			
	90.37				113.67				140.36				138.16				170.00			
	104.66				145.63				137.07				138.81				167.06			
	86.26	94.15	9.70	15.44	131.85	129.14	11.36	18.08	130.84	135.80	11.65	21.41	142.84	139.94	11.83	21.73	169.11	168.72	12.99	23.86
1.75	88.00				119.76				140.36				145.87				179.92			
	87.78				123.90				134.95				143.07				178.63			
	90.37				121.89				147.29				148.50				191.80			
	89.80	88.99	9.43	15.01	126.34	122.97	11.09	17.64	132.66	138.82	11.78	21.65	149.23	146.93	12.12	22.27	196.89	189.11	13.75	25.26
2	87.78				154.76				144.00				160.60				178.63			
	91.30				119.76				140.36				145.87				170.00			
	87.34				136.00				150.71				160.87				167.06			
	86.26	88.17	9.39	14.94	127.49	134.50	11.60	18.45	138.37	143.36	11.97	22.00	167.06	157.93	12.57	23.09	185.55	174.20	13.20	24.25
2.25	91.30				130.44				154.24				154.24				179.92			
	88.00				154.76				144.00				160.60				178.63			
	97.32				140.58				148.99				155.28				191.80			
	88.78	91.35	9.56	15.21	136.64	140.61	11.86	18.87	159.51	151.68	12.32	22.63	165.62	160.50	12.67	23.27	196.89	189.11	13.75	25.26
2.5	88.00				134.95				152.97				136.64				182.21			
	87.56				130.44				154.24				154.24				179.92			
	92.01				140.58				156.32				153.22				193.60			
	85.72	88.32	9.40	14.95	125.58	132.89	11.53	18.34	156.58	155.03	12.45	22.87	159.78	155.75	12.48	22.93	181.55	185.02	13.60	24.99
2.75	86.04				136.64				160.32				153.22				179.60			
	87.56				134.95				152.97				136.64				182.21			
	90.49				132.05				167.35				158.44				184.87			
	90.72	88.70	9.42	14.98	143.30	136.74	11.69	18.60	153.22	158.47	12.59	23.13	165.91	153.66	12.40	22.77	179.60	182.23	13.50	24.80
3	87.56				138.16				162.52				188.99				183.53			
	88.11				136.64				160.32				153.22				179.60			
	94.18				139.25				150.21				177.04				188.99			
	93.32	90.79	9.53	15.16	138.81	138.22	11.76	18.70	153.48	156.63	12.52	22.99	160.87	163.71	12.79	23.51	178.95	182.51	13.51	24.82
3.25	88.11				139.91				163.92				162.25				188.64			
	92.13				138.16				162.52				163.64				183.53			
	91.89				134.11				147.29				168.52				196.52			
	91.89	91.01	9.54	15.18	151.96	141.03	11.88	18.89	155.80	157.38	12.55	23.05	167.35	166.50	12.90	23.71	194.69	191.58	13.84	25.43
3.5	92.13				138.59				135.79				168.23				197.26			
	81.62				139.91				163.92				162.25				188.64			
	85.62				134.53				160.05				180.90				192.88			
	90.03	87.35	9.35	14.87	152.97	141.50	11.90	18.93	164.49	156.06	12.49	22.95	174.54	172.56	13.14	24.13	205.70	195.74	13.99	25.70
3.75	81.62				138.59				155.80				183.20				192.52			
	96.68				138.59				135.79				168.23				197.26			
	92.72				142.39				154.50				180.24				204.91			
	82.02	88.26	9.39	14.95	142.39	140.49	11.85	18.86	169.40	153.87	12.40	22.79	176.41	174.96	13.23	24.30	194.69	198.95	14.11	25.91
4	96.68				150.96				167.93				179.28				201.41			
	100.89				138.59				155.80				183.20				192.52			
	96.42				149.72				162.52				176.73				188.99			
	97.32	97.83	9.89	15.74	153.73	148.25	12.18	19.37	157.64	160.97	12.69	23.31	172.10	177.34	13.32	24.46	190.39	190.63	13.81	25.37
4.25	100.89				157.90				169.70				169.40				188.64			
	97.45				150.96				167.93				179.28				201.41			
	91.30				143.76				167.06				177.04				203.73			
	98.22	96.97	9.85	15.67	149.48	150.52	12.27	19.52	172.70	169.35	13.01	23.91	160.60	172.31	13.13	24.12	170.29	191.81	13.85	25.44
4.5	97.45				150.46				158.44				171.19				201.03			
	103.94				157.90				169.70				169.40				188.64			
	88.90				156.06				172.70				191.09				201.41			
	98.62	97.23	9.86	15.69	165.34	157.44	12.55	19.96	171.79	168.16	12.97	23.82	180.24	180.25	13.43	24.66	222.48	204.18	14.29	26.25
4.75	103.94				151.21				177.36				179.92				223.82			
	97.45				150.46				158.44				171.19				205.30			
	91.19				150.96				179.60				175.47				215.10			
	84.56	94.29	9.71	15.45	163.36	154.00	12.41	19.74	174.23	172.41	13.13	24.12	185.55	177.41	13.32	24.47	208.09	209.50	14.47	26.59
5	110.34				142.39				165.91				183.20				249.13			
	103.94				151.21				177.36				185.21				223.82			
	104.52				172.70				191.09				183.20				213.84			
	99.41	104.55	10.23	16.27	165.62	157.98	12.57	20.00	192.16	181.63	13.48	24.76	178.95	182.46	13.51	24.82	211.76	216.47	14.71	27.03

**Table 2:** Dataset for Vickers microhardness variations with the equivalent strain for the recycled Al–10%Si–Cu (Plots in Fig. 10).

r(mm)	Without HPT (HV)	1/4 turn		1/2 turn		1 turn		10 turns	
		HV	Equivalent strain	HV	Equivalent strain	HV	Equivalent strain	HV	Equivalent strain
−5	96.67	161.76	−5.34	168.24	−10.67	178.19	−21.34	214.07	−213.42
−4.75	93.34	156.59	−5.07	159.63	−10.14	187.43	−20.27	184.63	−202.75
−4.5	100.04	157.58	−4.80	161.80	−9.60	182.79	−19.21	193.55	−192.07
−4.25	103.24	151.33	−4.54	156.84	−9.07	181.01	−18.14	191.99	−181.40
−4	93.47	149.89	−4.27	154.80	−8.54	176.63	−17.07	184.50	−170.73
−3.75	93.54	144.05	−4.00	152.35	−8.00	172.13	−16.01	185.21	−160.06
−3.5	97.00	137.38	−3.73	149.56	−7.47	173.06	−14.94	182.64	−149.39
−3.25	96.81	139.98	−3.47	148.24	−6.94	167.51	−13.87	197.31	−138.72
−3	95.16	132.34	−3.20	148.89	−6.40	169.15	−12.80	190.72	−128.05
−2.75	89.94	141.70	−2.93	151.12	−5.87	162.84	−11.74	192.17	−117.38
−2.5	94.96	137.69	−2.67	153.38	−5.34	169.05	−10.67	191.50	−106.71
−2.25	91.56	132.06	−2.40	149.60	−4.80	167.82	−9.60	188.79	−96.04
−2	92.23	131.23	−2.13	148.75	−4.27	166.46	−8.54	187.90	−85.37
−1.75	93.76	124.18	−1.87	139.38	−3.73	165.85	−7.47	189.57	−74.70
−1.5	98.74	122.12	−1.60	127.26	−3.20	159.43	−6.40	188.67	−64.02
−1.25	90.60	121.48	−1.33	125.69	−2.67	150.39	−5.34	186.29	−53.35
−1	90.94	121.00	−1.07	123.00	−2.13	146.20	−4.27	183.54	−42.68
−0.75	91.60	114.11	−0.80	118.32	−1.60	131.94	−3.20	183.82	−32.01
−0.5	94.15	116.08	−0.53	124.68	−1.07	127.54	−2.13	180.94	−21.34
−0.25	91.60	109.15	−0.27	110.14	−0.53	122.94	−1.07	173.33	−10.67
0	93.32	108.44	0.00	108.93	0.00	120.39	0.00	173.09	0.00
0.25	90.90	110.04	0.27	104.35	0.53	117.51	1.07	179.69	10.67
0.5	91.88	114.25	0.53	107.43	1.07	117.51	2.13	174.49	21.34
0.75	87.00	115.48	0.80	121.47	1.60	116.29	3.20	167.54	32.01
1	93.15	118.69	1.07	126.71	2.13	129.07	4.27	171.50	42.68
1.25	103.08	128.76	1.33	132.15	2.67	136.93	5.34	167.58	53.35
1.5	94.15	129.14	1.60	135.80	3.20	139.94	6.40	168.72	64.02
1.75	88.99	122.97	1.87	138.82	3.73	146.93	7.47	189.11	74.70
2	88.17	134.50	2.13	143.36	4.27	157.93	8.54	174.20	85.37
2.25	91.35	140.61	2.40	151.68	4.80	160.50	9.60	189.11	96.04
2.5	88.32	132.89	2.67	155.03	5.34	155.75	10.67	185.02	106.71
2.75	88.70	136.74	2.93	158.47	5.87	153.66	11.74	182.23	117.38
3	90.79	138.22	3.20	156.63	6.40	163.71	12.80	182.51	128.05
3.25	91.01	141.03	3.47	157.38	6.94	166.50	13.87	191.58	138.72
3.5	87.35	141.50	3.73	156.06	7.47	172.56	14.94	195.74	149.39
3.75	88.26	140.49	4.00	153.87	8.00	174.96	16.01	198.95	160.06
4	97.83	148.25	4.27	160.97	8.54	177.34	17.07	190.63	170.73
4.25	96.97	150.52	4.54	169.35	9.07	172.31	18.14	191.81	181.40
4.5	97.23	157.44	4.80	168.16	9.60	180.25	19.21	204.18	192.07
4.75	94.29	154.00	5.07	172.41	10.14	177.41	20.27	209.50	202.75
5	104.55	157.98	5.34	181.63	10.67	182.46	21.34	216.47	213.42

## 2 Experimental design, materials, and methods

The recycled Al–Si piston alloy used in this research had the following composition: 10.6%Si, 1.36%Cu, 1.08%Ni, 0.78%Mg, 1.06%Fe, 0.08%Mn, 0.03%Cr, 0.06%Ti, 0.02%Sn, 0.09%Zn and 0.04%K. The alloy was produced through sand casting. Specimens of the recycled cast Al–Si piston alloys were sliced from the as-received ingots with a diameter of 10 mm and ground with abrasive papers to a thickness of 0.8–0.85 mm. The HPT processing was conducted at room temperature at 1rpm rotation of the lower anvil and under a pressure of 3.0 GPa of the upper anvil on an HPT facility shown in Fig. 1. Briefly, the HPT facility consists of high-strength tool steel lower and upper anvils with circular cavities of 10 mm diameter and 0.25 mm thickness. The specimens were processed for 1/4, 1/2 1 and 10 turns of high-pressure torsion processing.

Samples from the as-received alloy and the HPT-processed specimens were mounted and ground using silicon carbide papers up to #1200. They were then polished up to 1/4 μm mirror-like finish using diamond pastes. Finally, the specimens were polished to oxide polish suspension finish (0.05 m). The microstructural observations were undertaken using optical and scanning electron microscopy. Identification of the intermetallic phases in the alloy was carried out using the Electron diffraction Spectrometer (EDS) incorporated into the SEM equipment. The micrographs are presented in Fig. 2, Fig. 3, Fig. 4, Fig. 5, Fig. 6.

The images were also analyzed using ImageJ software to quantify the microstructural refinement during the HPT process. From the analysis, the area (A) and circularity of particles were obtained. Circularity (C) is a measure of shape which is calculated as function of area (A) and perimeter (P) of particles as follows:

$$\frac{4A\pi }{P^{2}}$$

The plots for particle circularity against the area of the particles at 1/4 and 10 turns at the central regions and edges of the samples are presented in Fig. 7.

**Fig. 8:**
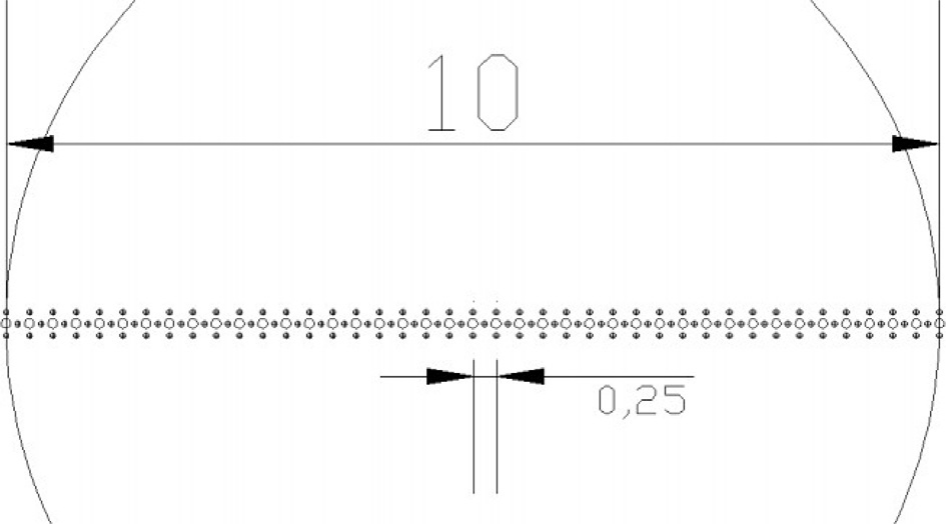
Scheme of the microhardness measurement on HPT samples.

**Fig. 9:**
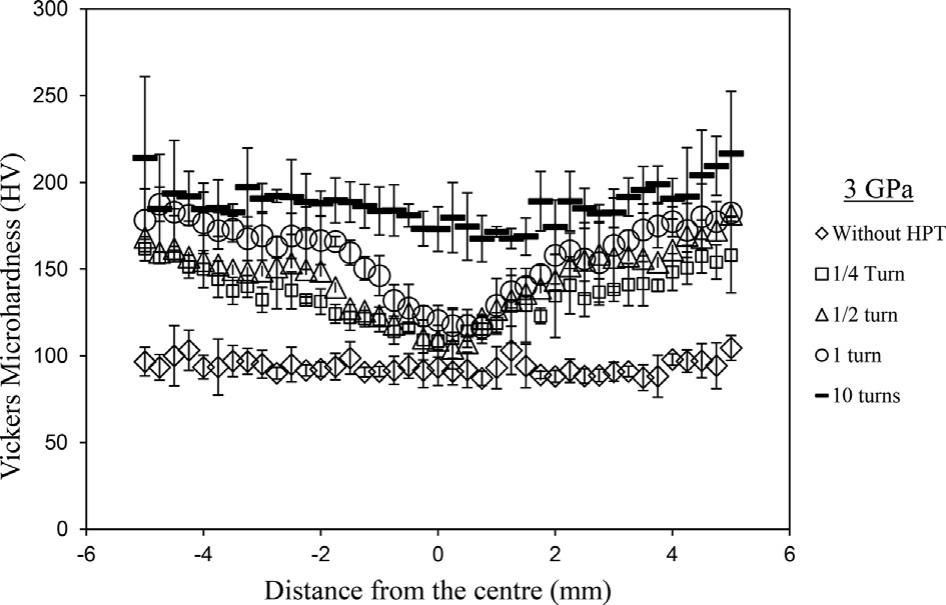
The average Vickers microhardness (Hv) against the distance from the center of the disk for 1/4, 1/2, 1 and 10 turns of HPT processing at a pressure of 3 GPa on the upper die and at a rotational speed of 1rpm of lower die. The microhardness line profile for the as received sample is also shown for comparison. The error bars computed using 95% confidence level are also shown.

Microhardness measurements were then taken along the diameter of the polished unprocessed and HPT-processed specimens. A load of 300 g for a dwell time of 10 s was used and the procedure for microhardness measurements is illustrated in Fig. 8. The microhardness plots along the radius of the samples are shown in Fig. 9. The strain variation across the surface of the HPT samples were computed as described in references [1], [2], [3] and presented in Fig. 10.

## Appendix A Supplementary data

Table SEQ Table ∖* ARABIC 1. Data for area and circularity of particles at the central and edge regions of the HPT processed sample (Plots in Fig. 7)
