# Supplementary material for: Data on the effect of high-pressure torsion processing on secondary cast Al–10%Si– Cu piston alloy: Methods, microstructure and mechanical characterizations
Source: Data Brief. 2019 Jul 3;25:104160. doi: 10.1016/j.dib.2019.104160 (PMC6700338; doi:10.1016/j.dib.2019.104160)
Supplement: Supplementary file 1 — Table SEQ Table ∖* ARABIC 1. Data for area and circularity of particles at the central and edge regions of the HPT processed sample (Plots in Fig. 7) [file mmc1.docx]

Table 1. Data for area and circularity of particles at the central and edge regions of the HPT processed sample (Plots in Fig. 7)

| **1/4 turn** | | | | **10 turns** | | | |
| --- | --- | --- | --- | --- | --- | --- | --- |
| **Central region** | | **Edge** | | **Central region** | | **Edge** | |
| **Area** | **Circularity** | **Area** | **Circularity** | **Area** | **Circularity** | **Area** | **Circularity** |
| 105.52 | 0.06 | 0.04 | 1.00 | 17.36 | 0.59 | 287.87 | 0.05 |
| 0.04 | 1.00 | 108.31 | 0.44 | 23.15 | 0.62 | 19.70 | 0.29 |
| 1.23 | 0.15 | 7.00 | 0.76 | 14.80 | 0.53 | 5.03 | 0.32 |
| 0.15 | 0.59 | 125.27 | 0.41 | 71.55 | 0.39 | 2.22 | 0.55 |
| 1.20 | 0.30 | 19.75 | 0.51 | 10.17 | 0.33 | 0.95 | 0.86 |
| 0.10 | 1.00 | 159.32 | 0.17 | 14.54 | 0.67 | 2.14 | 0.74 |
| 0.14 | 0.56 | 1.97 | 0.46 | 12.75 | 0.67 | 144.46 | 0.12 |
| 0.06 | 0.97 | 87.64 | 0.41 | 1.50 | 0.69 | 35.46 | 0.31 |
| 0.04 | 1.00 | 45.29 | 0.28 | 98.63 | 0.26 | 5.88 | 0.50 |
| 0.06 | 0.97 | 66.55 | 0.44 | 32.25 | 0.47 | 5.98 | 0.84 |
| 7.95 | 0.70 | 3.55 | 0.77 | 134.26 | 0.29 | 26.12 | 0.29 |
| 4.53 | 0.82 | 39.58 | 0.41 | 11.00 | 0.55 | 1.56 | 0.70 |
| 578.55 | 0.17 | 11.15 | 0.78 | 125.77 | 0.68 | 3.67 | 0.73 |
| 65.03 | 0.34 | 0.10 | 1.00 | 75.23 | 0.17 | 6.71 | 0.83 |
| 11.42 | 0.35 | 44.62 | 0.62 | 653.09 | 0.60 | 3.14 | 0.63 |
| 121.89 | 0.36 | 11.00 | 0.63 | 29.71 | 0.26 | 10.67 | 0.43 |
| 2.87 | 0.66 | 0.27 | 0.70 | 1.39 | 0.85 | 6.89 | 0.71 |
| 163.85 | 0.26 | 9.92 | 0.87 | 14.66 | 0.88 | 0.04 | 1.00 |
| 12.64 | 0.83 | 32.95 | 0.29 | 13.41 | 0.67 | 2.28 | 0.62 |
| 0.10 | 0.76 | 0.46 | 1.00 | 0.12 | 0.92 | 0.08 | 0.74 |
| 2.55 | 0.48 | 0.04 | 1.00 | 0.29 | 0.46 | 57.08 | 0.37 |
| 0.04 | 1.00 | 51.87 | 0.71 | 0.10 | 1.00 | 0.04 | 1.00 |
| 0.69 | 0.31 | 2.33 | 0.75 | 0.17 | 0.48 | 0.68 | 0.69 |
| 1.76 | 0.12 | 3.24 | 0.93 | 0.10 | 0.44 | 311.36 | 0.07 |
| 98.78 | 0.08 | 16.88 | 0.86 | 0.25 | 0.65 | 5.02 | 0.58 |
| 13.66 | 0.42 | 48.26 | 0.56 | 0.12 | 1.00 | 0.52 | 0.84 |
| 0.08 | 0.70 | 0.12 | 1.00 | 7.72 | 0.76 | 32.99 | 0.25 |
| 0.85 | 0.25 | 0.75 | 1.00 | 0.08 | 0.70 | 0.39 | 0.90 |
| 0.14 | 0.80 | 0.60 | 1.00 | 0.08 | 0.70 | 396.20 | 0.26 |
| 0.14 | 0.69 | 1.83 | 0.96 | 1.06 | 0.63 | 0.04 | 1.00 |
| 0.08 | 0.54 | 3.38 | 0.79 | 0.04 | 1.00 | 0.15 | 0.71 |
| 0.04 | 1.00 | 26.41 | 0.68 | 182.21 | 0.65 | 29.69 | 0.34 |
| 0.04 | 1.00 | 3.05 | 0.88 | 26.23 | 0.70 | 0.37 | 0.45 |
| 0.04 | 0.79 | 4.80 | 0.82 | 0.04 | 1.00 | 1.74 | 0.70 |
| 0.06 | 1.00 | 99.67 | 0.25 | 21.64 | 0.56 | 0.04 | 1.00 |
| 0.08 | 0.86 | 306.56 | 0.15 | 22.34 | 0.74 | 0.35 | 0.73 |
| 0.04 | 1.00 | 47.49 | 0.52 | 3.86 | 0.84 | 0.91 | 0.99 |
| 0.04 | 1.00 | 0.98 | 0.75 | 3.88 | 0.78 | 0.52 | 0.73 |
| 0.04 | 0.79 | 109.76 | 0.48 | 2.57 | 0.85 | 0.27 | 0.63 |
| 0.06 | 1.00 | 108.41 | 0.29 | 0.54 | 1.00 | 0.17 | 1.00 |
| 0.06 | 1.00 | 2.87 | 0.72 | 2.30 | 0.91 | 2.45 | 0.81 |
| 0.06 | 1.00 | 2.08 | 0.95 | 3.32 | 0.84 | 0.12 | 1.00 |
| 0.50 | 0.59 | 1.16 | 0.57 | 1.66 | 0.85 | 0.08 | 0.86 |
| 0.04 | 1.00 | 0.04 | 1.00 | 0.50 | 0.74 | 0.48 | 1.00 |
| 0.08 | 0.61 | 23.13 | 0.52 | 1.62 | 0.96 | 0.04 | 1.00 |
| 0.04 | 1.00 | 0.06 | 1.00 | 7.54 | 0.73 | 0.31 | 0.77 |
| 0.06 | 1.00 | 6.87 | 0.83 | 3.63 | 0.74 | 0.15 | 0.74 |
| 0.10 | 1.00 | 103.65 | 0.24 | 0.56 | 1.00 | 11.79 | 0.75 |
| 0.04 | 0.79 | 48.07 | 0.68 | 0.23 | 1.00 | 14.04 | 0.29 |
| 0.12 | 1.00 | 29.30 | 0.66 | 87.02 | 0.72 | 0.39 | 0.72 |
| 0.04 | 0.79 | 18.77 | 0.64 | 26.04 | 0.50 | 73.92 | 0.30 |
| 0.04 | 1.00 | 16.22 | 0.70 | 1.31 | 0.88 | 24.54 | 0.33 |
| 0.06 | 1.00 | 2.97 | 0.78 | 25.73 | 0.63 | 0.04 | 1.00 |
| 0.14 | 1.00 | 0.83 | 0.67 | 1.45 | 0.51 | 1.49 | 0.81 |
| 0.77 | 0.33 | 6.37 | 0.40 | 0.08 | 1.00 | 2.35 | 0.65 |
| 0.04 | 1.00 | 0.37 | 0.94 | 0.14 | 1.00 | 0.81 | 0.81 |
| 0.04 | 1.00 | 2.85 | 0.95 | 4.28 | 0.54 | 45.64 | 0.35 |
| 0.12 | 0.92 | 58.95 | 0.80 | 72.43 | 0.32 | 6.00 | 0.75 |
| 0.27 | 0.36 | 0.04 | 1.00 | 0.60 | 0.96 | 5.83 | 0.72 |
| 0.04 | 1.00 | 1.43 | 0.76 | 0.54 | 0.94 | 0.56 | 1.00 |
| 0.04 | 1.00 | 4.07 | 0.61 | 1.02 | 0.93 | 0.42 | 0.79 |
| 0.04 | 0.79 | 0.15 | 1.00 | 5.63 | 0.83 | 0.19 | 0.81 |
| 0.04 | 0.79 | 5.27 | 0.78 | 0.27 | 0.81 | 0.25 | 0.85 |
| 0.06 | 1.00 | 0.04 | 1.00 | 3.78 | 0.89 | 1.00 | 0.90 |
| 0.12 | 0.59 | 29.32 | 0.42 | 0.60 | 0.84 | 0.14 | 0.80 |
| 0.08 | 1.00 | 45.35 | 0.62 | 35.69 | 0.63 | 0.08 | 0.86 |
| 0.06 | 1.00 | 0.12 | 0.62 | 2.93 | 0.88 | 0.15 | 1.00 |
| 0.06 | 0.64 | 0.31 | 1.00 | 0.12 | 0.62 | 1.04 | 0.93 |
| 0.08 | 1.00 | 60.13 | 0.33 | 69.93 | 0.49 | 45.97 | 0.38 |
| 0.04 | 1.00 | 8.99 | 0.80 | 0.69 | 0.88 | 0.12 | 1.00 |
| 0.10 | 1.00 | 0.68 | 0.78 | 10.13 | 0.56 | 0.06 | 0.52 |
| 0.04 | 1.00 | 0.66 | 0.65 | 16.34 | 0.77 | 0.14 | 1.00 |
| 0.06 | 1.00 | 0.44 | 0.52 | 6.79 | 0.34 | 0.96 | 0.90 |
| 0.08 | 1.00 | 6.94 | 0.72 | 83.55 | 0.48 | 0.81 | 1.00 |
| 0.10 | 1.00 | 1.20 | 0.88 | 5.98 | 0.53 | 0.15 | 0.91 |
| 0.08 | 0.86 | 1.83 | 0.95 | 0.48 | 0.92 | 0.06 | 0.81 |
| 0.06 | 1.00 | 0.50 | 0.49 | 15.28 | 0.68 | 0.23 | 0.46 |
| 0.04 | 0.79 | 0.14 | 0.80 | 73.51 | 0.76 | 1.39 | 0.81 |
| 0.08 | 1.00 | 84.76 | 0.28 | 4.76 | 0.67 | 0.58 | 0.93 |
| 0.41 | 0.47 | 42.63 | 0.54 | 20.41 | 0.60 | 12.31 | 0.44 |
| 0.25 | 0.27 | 231.15 | 0.29 | 32.08 | 0.88 | 0.17 | 0.92 |
| 0.06 | 1.00 | 47.78 | 0.50 | 1.52 | 0.84 | 0.15 | 1.00 |
| 63.35 | 0.68 | 0.04 | 1.00 | 0.04 | 1.00 | 8.89 | 0.73 |
| 0.06 | 0.81 | 1.10 | 0.98 | 128.40 | 0.47 | 16.17 | 0.54 |
| 0.19 | 0.29 | 7.95 | 0.60 | 10.46 | 0.46 | 1.12 | 0.82 |
| 0.12 | 0.53 | 321.93 | 0.14 | 5.29 | 0.79 | 0.42 | 0.79 |
| 0.04 | 1.00 | 0.23 | 0.93 | 73.01 | 0.50 | 1.77 | 0.42 |
| 0.08 | 0.39 | 0.21 | 0.98 | 18.62 | 0.48 | 11.59 | 0.67 |
| 0.19 | 0.54 | 2.37 | 0.82 | 0.17 | 0.88 | 2.10 | 0.79 |
| 1.83 | 0.20 | 2.31 | 0.84 | 33.24 | 0.60 | 2.16 | 0.61 |
| 0.21 | 0.66 | 125.23 | 0.29 | 0.35 | 0.87 | 0.06 | 0.81 |
| 0.04 | 0.79 | 46.95 | 0.41 | 43.31 | 0.42 | 0.10 | 0.87 |
| 0.12 | 1.00 | 148.94 | 0.28 | 0.31 | 0.86 | 1.35 | 0.37 |
| 0.33 | 0.53 | 2.04 | 0.67 | 21.91 | 0.71 | 0.10 | 1.00 |
| 0.23 | 0.44 | 0.12 | 1.00 | 1.00 | 0.90 | 135.69 | 0.09 |
| 0.04 | 1.00 | 0.77 | 0.77 | 3.86 | 0.71 | 0.10 | 1.00 |
| 1.79 | 0.26 | 1.50 | 0.60 | 19.64 | 0.81 | 3.47 | 0.52 |
| 0.10 | 0.67 | 2.30 | 0.75 | 0.04 | 0.79 | 0.42 | 0.34 |
| 0.04 | 0.79 | 15.32 | 0.50 | 1.85 | 0.81 | 0.04 | 1.00 |
| 0.04 | 0.79 | 146.39 | 0.36 | 0.08 | 0.86 | 60.38 | 0.16 |
| 0.08 | 0.70 | 43.23 | 0.39 | 0.48 | 0.64 | 0.12 | 1.00 |
| 0.06 | 0.81 | 24.90 | 0.54 | 42.90 | 0.59 | 0.06 | 0.64 |
| 0.06 | 0.64 | 0.48 | 1.00 | 0.42 | 0.92 | 3.63 | 0.74 |
| 0.04 | 0.79 | 14.14 | 0.65 | 38.37 | 0.72 | 0.64 | 0.94 |
| 0.06 | 1.00 | 1.35 | 0.83 | 1.37 | 0.99 | 3.34 | 0.71 |
| 0.04 | 1.00 | 18.65 | 0.41 | 13.87 | 0.78 | 35.46 | 0.39 |
| 0.15 | 0.57 | 0.23 | 0.54 | 0.85 | 0.85 | 4.59 | 0.75 |
| 0.10 | 0.67 | 0.04 | 1.00 | 16.65 | 0.85 | 0.12 | 0.81 |
| 0.04 | 0.79 | 0.04 | 1.00 | 1.02 | 0.81 | 2.95 | 0.88 |
| 0.04 | 1.00 | 0.35 | 0.75 | 0.23 | 1.00 | 0.14 | 0.90 |
| 0.68 | 0.26 | 34.76 | 0.81 | 2.41 | 0.89 | 1.29 | 0.79 |
| 0.04 | 1.00 | 46.37 | 0.52 | 16.99 | 0.83 | 0.15 | 1.00 |
| 2.37 | 0.81 | 6.31 | 0.44 | 1.99 | 0.86 | 0.23 | 0.78 |
| 0.17 | 0.64 | 5.27 | 0.68 | 24.87 | 0.72 | 2.18 | 0.52 |
| 0.17 | 0.54 | 2.58 | 0.76 | 65.88 | 0.27 | 0.93 | 0.74 |
| 0.04 | 1.00 | 137.87 | 0.46 | 0.15 | 0.91 | 0.27 | 0.91 |
| 0.44 | 0.71 | 21.35 | 0.41 | 61.90 | 0.39 | 0.21 | 1.00 |
| 0.14 | 0.51 | 19.25 | 0.81 | 0.52 | 1.00 | 0.17 | 0.73 |
| 0.06 | 1.00 | 2.53 | 0.85 | 0.79 | 0.98 | 0.06 | 1.00 |
| 0.19 | 0.60 | 2.97 | 0.83 | 15.64 | 0.78 | 11.63 | 0.46 |
| 0.04 | 1.00 | 3.11 | 0.55 | 1.43 | 0.92 | 0.25 | 0.92 |
| 0.12 | 0.39 | 17.71 | 0.77 | 0.77 | 0.81 | 0.19 | 0.81 |
| 0.04 | 0.79 | 65.32 | 0.32 | 1.08 | 0.95 | 0.81 | 0.74 |
| 0.10 | 0.67 | 0.04 | 1.00 | 0.12 | 1.00 | 0.15 | 0.91 |
| 0.04 | 1.00 | 6.46 | 0.77 | 1.06 | 0.95 | 0.27 | 1.00 |
| 0.06 | 1.00 | 238.35 | 0.67 | 0.14 | 0.94 | 6.87 | 0.37 |
| 0.12 | 0.92 | 5.23 | 0.72 | 0.08 | 1.00 | 0.21 | 0.98 |
| 0.12 | 0.81 | 0.21 | 0.98 | 3.05 | 0.76 | 12.89 | 0.36 |
| 0.06 | 1.00 | 99.52 | 0.55 | 1.99 | 0.68 | 3.05 | 0.58 |
| 0.10 | 0.87 | 4.57 | 0.82 | 3.82 | 0.89 | 0.48 | 1.00 |
| 0.04 | 0.79 | 51.33 | 0.37 | 28.80 | 0.64 | 0.75 | 0.93 |
| 0.06 | 1.00 | 0.04 | 0.79 | 19.62 | 0.60 | 0.41 | 0.88 |
| 1.70 | 0.79 | 0.42 | 0.79 | 77.47 | 0.74 | 0.41 | 0.73 |
| 0.15 | 0.91 | 8.39 | 0.88 | 0.44 | 0.96 | 0.23 | 0.85 |
| 0.14 | 0.50 | 15.99 | 0.71 | 0.56 | 0.98 | 16.24 | 0.54 |
| 14.16 | 0.72 | 8.78 | 0.81 | 1.08 | 0.84 | 0.95 | 0.80 |
| 0.12 | 0.92 | 4.78 | 0.81 | 0.46 | 1.00 | 0.46 | 0.79 |
| 0.04 | 0.79 | 3.74 | 0.76 | 2.22 | 0.83 | 0.52 | 0.84 |
| 0.14 | 0.62 | 6.15 | 0.81 | 12.33 | 0.61 | 0.79 | 0.87 |
| 0.04 | 0.79 | 102.74 | 0.24 | 0.25 | 0.85 | 0.27 | 0.91 |
| 8.85 | 0.58 | 9.66 | 0.34 | 0.25 | 0.82 | 5.59 | 0.65 |
| 0.06 | 1.00 | 28.18 | 0.50 | 6.71 | 0.86 | 34.22 | 0.15 |
| 0.06 | 1.00 | 99.23 | 0.23 | 10.94 | 0.61 | 17.40 | 0.26 |
| 0.14 | 0.90 | 22.63 | 0.70 | 6.42 | 0.73 | 1.39 | 0.70 |
| 0.08 | 0.51 | 160.36 | 0.39 | 0.79 | 0.48 | 0.27 | 0.99 |
| 0.04 | 0.79 | 103.72 | 0.51 | 3.90 | 0.68 | 0.15 | 1.00 |
| 0.06 | 0.97 | 0.81 | 0.95 | 0.04 | 1.00 | 3.28 | 0.76 |
| 1.41 | 0.18 | 0.19 | 1.00 | 24.36 | 0.80 | 0.50 | 0.79 |
| 0.06 | 0.52 | 3.92 | 0.78 | 2.55 | 0.82 | 0.64 | 0.94 |
| 0.06 | 1.00 | 16.84 | 0.81 | 3.13 | 0.94 | 0.14 | 0.94 |
| 6.77 | 0.75 | 40.72 | 0.32 | 2.03 | 0.76 | 0.93 | 0.97 |
| 0.08 | 0.86 | 0.10 | 1.00 | 76.81 | 0.27 | 0.25 | 0.75 |
| 0.04 | 1.00 | 99.59 | 0.65 | 0.04 | 1.00 | 0.77 | 0.85 |
| 0.04 | 1.00 | 20.54 | 0.70 | 10.22 | 0.57 | 0.48 | 0.95 |
| 0.25 | 0.50 | 0.68 | 0.69 | 1.83 | 0.96 | 2.24 | 0.88 |
| 0.04 | 0.79 | 16.65 | 0.72 | 0.48 | 0.90 | 0.08 | 1.00 |
| 0.04 | 0.79 | 143.25 | 0.39 | 2.76 | 0.46 | 2.97 | 0.81 |
| 0.10 | 1.00 | 3.43 | 0.88 | 2.66 | 0.83 | 0.04 | 0.79 |
| 1.50 | 0.60 | 8.06 | 0.87 | 230.83 | 0.10 | 0.08 | 0.86 |
| 0.06 | 0.97 | 28.13 | 0.81 | 4.40 | 0.67 | 2.18 | 0.86 |
| 0.29 | 1.00 | 5.83 | 0.41 | 3.28 | 0.78 | 0.21 | 0.89 |
| 0.06 | 0.97 | 100.35 | 0.32 | 1.62 | 0.96 | 0.08 | 0.86 |
| 0.06 | 0.52 | 1.68 | 0.92 | 2.76 | 0.88 | 0.04 | 1.00 |
| 0.17 | 0.88 | 3.07 | 0.82 | 3.38 | 0.70 | 1.41 | 0.72 |
| 0.04 | 1.00 | 0.04 | 1.00 | 17.65 | 0.79 | 0.19 | 0.98 |
| 0.04 | 1.00 | 0.68 | 0.59 | 12.79 | 0.74 | 31.12 | 0.30 |
| 0.06 | 1.00 | 1.39 | 0.59 | 15.16 | 0.72 | 0.31 | 1.00 |
| 0.10 | 0.51 | 1.20 | 0.88 | 138.21 | 0.33 | 0.46 | 0.92 |
| 0.19 | 0.81 | 32.12 | 0.55 | 9.49 | 0.76 | 32.27 | 0.80 |
| 0.08 | 0.54 | 0.71 | 0.63 | 24.81 | 0.76 | 1.50 | 0.86 |
| 0.14 | 0.94 | 8.10 | 0.44 | 0.71 | 0.95 | 1.58 | 0.48 |
| 0.29 | 0.90 | 0.06 | 0.75 | 0.08 | 0.86 | 0.12 | 1.00 |
| 0.08 | 0.64 | 3.97 | 0.87 | 1.60 | 0.88 | 4.26 | 0.89 |
| 0.10 | 0.87 | 18.67 | 0.45 | 38.19 | 0.57 | 2.76 | 0.63 |
| 0.08 | 0.51 | 5.50 | 0.87 | 5.25 | 0.60 | 0.42 | 0.99 |
| 0.06 | 0.97 | 0.33 | 1.00 | 20.89 | 0.82 | 0.14 | 1.00 |
| 1.23 | 0.25 | 1.41 | 0.56 | 2.58 | 0.75 | 83.74 | 0.16 |
| 89.27 | 0.59 | 16.76 | 0.37 | 0.77 | 0.95 | 0.68 | 0.95 |
| 0.37 | 0.32 | 1.99 | 0.89 | 0.77 | 0.71 | 0.06 | 1.00 |
| 0.04 | 1.00 | 185.86 | 0.34 | 6.21 | 0.79 | 0.35 | 0.75 |
| 0.10 | 0.87 | 0.08 | 1.00 | 0.08 | 1.00 | 1.87 | 0.94 |
| 0.04 | 1.00 | 13.21 | 0.82 | 29.92 | 0.58 | 0.62 | 0.97 |
| 0.04 | 1.00 | 1.10 | 0.82 | 0.31 | 0.86 | 10.57 | 0.44 |
| 0.31 | 0.41 | 53.59 | 0.35 | 37.50 | 0.81 | 0.08 | 0.86 |
| 0.04 | 0.79 | 4.84 | 0.78 | 0.08 | 1.00 | 0.31 | 1.00 |
| 0.12 | 0.77 | 48.17 | 0.79 | 4.11 | 0.83 | 1.54 | 0.72 |
| 0.10 | 0.87 | 8.91 | 0.73 | 24.29 | 0.48 | 0.93 | 0.50 |
| 0.04 | 0.79 | 13.77 | 0.67 | 0.27 | 1.00 | 7.97 | 0.80 |
| 0.04 | 1.00 | 22.69 | 0.81 | 1.10 | 0.81 | 32.25 | 0.26 |
| 0.08 | 1.00 | 1.45 | 0.89 | 0.39 | 0.45 | 57.54 | 0.22 |
| 0.04 | 0.79 | 3.47 | 0.50 | 0.41 | 0.88 | 0.60 | 0.91 |
| 224.92 | 0.13 | 0.31 | 1.00 | 2.43 | 0.92 | 0.10 | 1.00 |
| 0.17 | 0.80 | 0.73 | 0.69 | 166.90 | 0.17 | 0.04 | 1.00 |
| 0.04 | 0.79 | 0.08 | 0.86 | 0.08 | 1.00 | 2.82 | 0.41 |
| 0.06 | 0.64 | 6.12 | 0.59 | 4.71 | 0.68 | 0.64 | 0.83 |
| 0.06 | 1.00 | 53.41 | 0.61 | 0.25 | 0.92 | 0.37 | 0.85 |
| 0.54 | 0.43 | 0.06 | 1.00 | 126.99 | 0.45 | 5.63 | 0.78 |
| 0.10 | 1.00 | 23.98 | 0.41 | 3.05 | 0.84 | 0.95 | 0.68 |
| 0.06 | 1.00 | 7.64 | 0.70 | 5.21 | 0.68 | 0.41 | 0.86 |
| 0.06 | 1.00 | 4.73 | 0.69 | 0.06 | 0.97 | 0.56 | 0.92 |
| 0.06 | 0.75 | 66.11 | 0.49 | 5.29 | 0.88 | 0.85 | 0.65 |
| 0.04 | 0.79 | 17.82 | 0.84 | 13.60 | 0.62 | 0.04 | 1.00 |
| 0.06 | 0.81 | 2.35 | 0.66 | 1.37 | 0.92 | 0.08 | 0.70 |
| 0.04 | 0.79 | 0.62 | 0.99 | 55.98 | 0.25 | 0.21 | 1.00 |
| 0.06 | 1.00 | 39.78 | 0.58 | 3.13 | 0.61 | 0.10 | 0.87 |
| 0.08 | 0.86 | 26.91 | 0.67 | 0.35 | 1.00 | 0.19 | 1.00 |
| 0.10 | 1.00 | 81.62 | 0.32 | 0.19 | 0.71 | 0.06 | 0.97 |
| 0.08 | 0.61 | 87.94 | 0.59 | 90.57 | 0.36 | 0.31 | 0.80 |
| 0.06 | 0.81 | 348.53 | 0.24 | 4.15 | 0.83 | 28.13 | 0.30 |
| 547.78 | 0.11 | 1.00 | 0.81 | 1.95 | 0.95 | 9.24 | 0.54 |
| 0.10 | 0.67 | 0.15 | 1.00 | 1.70 | 0.82 | 0.37 | 0.94 |
| 0.04 | 0.79 | 21.76 | 0.26 | 2.51 | 0.70 | 1.02 | 0.67 |
| 0.35 | 0.40 | 4.15 | 0.90 | 35.76 | 0.75 | 1.64 | 0.89 |
| 0.08 | 0.61 | 0.77 | 0.89 | 1.02 | 0.37 | 0.06 | 1.00 |
| 0.19 | 0.48 | 1.08 | 0.75 | 67.75 | 0.33 | 0.21 | 1.00 |
| 0.06 | 1.00 | 2.74 | 0.89 | 43.13 | 0.47 | 15.72 | 0.51 |
| 0.44 | 0.23 | 72.20 | 0.65 | 0.89 | 0.99 | 0.12 | 1.00 |
| 0.06 | 0.97 | 27.89 | 0.37 | 4.48 | 0.70 | 0.12 | 1.00 |
| 0.10 | 0.87 | 7.20 | 0.61 | 9.53 | 0.69 | 1.29 | 0.64 |
| 0.04 | 1.00 | 9.12 | 0.75 | 184.30 | 0.40 | 0.19 | 0.78 |
| 0.04 | 0.79 | 50.12 | 0.44 | 11.30 | 0.79 | 0.54 | 0.92 |
| 0.08 | 1.00 | 0.06 | 0.97 | 0.87 | 0.95 | 0.33 | 0.85 |
| 0.06 | 0.81 | 1.50 | 0.70 | 5.73 | 0.73 | 1.18 | 0.90 |
| 0.25 | 0.33 | 78.28 | 0.33 | 3.14 | 0.74 | 0.56 | 0.95 |
| 0.33 | 0.49 | 1.68 | 0.73 | 0.91 | 0.89 | 0.04 | 1.00 |
| 0.08 | 1.00 | 13.46 | 0.61 | 0.66 | 0.87 | 0.15 | 0.74 |
| 0.21 | 0.98 | 18.94 | 0.68 | 0.15 | 0.91 | 0.35 | 0.89 |
| 134.86 | 0.24 | 1.74 | 0.81 | 1.52 | 0.46 | 0.35 | 1.00 |
| 1.47 | 0.22 | 77.89 | 0.40 | 1.66 | 0.86 | 1.35 | 0.84 |
| 0.64 | 0.38 | 68.36 | 0.37 | 17.42 | 0.50 | 0.12 | 1.00 |
| 0.15 | 0.62 | 9.80 | 0.63 | 0.12 | 1.00 | 0.96 | 0.83 |
| 0.08 | 1.00 | 96.14 | 0.30 | 16.86 | 0.54 | 0.14 | 1.00 |
| 0.04 | 1.00 | 1.93 | 0.84 | 0.04 | 0.79 | 1.12 | 0.97 |
| 0.04 | 0.79 | 106.44 | 0.23 | 0.15 | 0.91 | 0.04 | 1.00 |
| 0.14 | 1.00 | 32.77 | 0.66 | 57.95 | 0.68 | 0.35 | 0.66 |
| 0.06 | 0.75 | 0.17 | 0.54 | 10.26 | 0.33 | 0.15 | 0.82 |
| 0.04 | 1.00 | 31.94 | 0.79 | 125.54 | 0.48 | 1.76 | 0.77 |
| 0.04 | 1.00 | 0.31 | 0.86 | 3.43 | 0.67 | 0.06 | 0.81 |
| 0.23 | 0.70 | 2.93 | 0.77 | 36.52 | 0.27 | 10.05 | 0.61 |
| 0.06 | 1.00 | 115.76 | 0.39 | 1.60 | 0.90 | 2.87 | 0.77 |
| 0.04 | 1.00 | 2.64 | 0.86 | 43.90 | 0.52 | 1.70 | 0.74 |
| 0.04 | 0.79 | 88.91 | 0.81 | 142.79 | 0.60 | 3.01 | 0.82 |
| 39.93 | 0.38 | 1.20 | 0.91 | 0.15 | 1.00 | 17.77 | 0.34 |
| 0.12 | 0.48 | 8.87 | 0.56 | 86.81 | 0.39 | 0.14 | 0.56 |
| 0.21 | 0.32 | 43.07 | 0.68 | 32.83 | 0.61 | 0.14 | 1.00 |
| 0.04 | 0.79 | 4.61 | 0.96 | 1.76 | 0.88 | 20.35 | 0.18 |
| 0.12 | 0.50 | 53.13 | 0.28 | 6.46 | 0.82 | 0.27 | 0.77 |
| 0.08 | 0.86 | 1.70 | 0.94 | 8.99 | 0.43 | 49.67 | 0.27 |
| 0.04 | 0.79 | 6.42 | 0.92 | 0.46 | 0.74 | 42.82 | 0.31 |
| 0.69 | 0.17 | 13.83 | 0.65 | 5.30 | 0.83 | 0.04 | 1.00 |
| 0.04 | 1.00 | 70.87 | 0.63 | 0.50 | 0.41 | 0.08 | 0.86 |
| 0.08 | 1.00 | 20.12 | 0.71 | 3.67 | 0.88 | 14.95 | 0.26 |
| 0.04 | 1.00 | 2.84 | 0.78 | 29.98 | 0.66 | 0.15 | 0.91 |
| 4.40 | 0.33 | 1.23 | 0.68 | 0.19 | 1.00 | 1.64 | 0.46 |
| 0.08 | 0.86 | 4.92 | 0.16 | 3.94 | 0.71 | 0.69 | 1.00 |
| 0.17 | 1.00 | 3.84 | 0.82 | 4.34 | 0.77 | 0.44 | 1.00 |
| 0.04 | 1.00 | 28.65 | 0.84 | 1.18 | 0.91 | 0.04 | 1.00 |
| 0.04 | 1.00 | 9.74 | 0.81 | 5.09 | 0.84 | 0.91 | 0.95 |
| 0.23 | 0.39 | 143.52 | 0.47 | 1.79 | 0.83 | 0.58 | 0.99 |
| 0.04 | 1.00 | 25.41 | 0.25 | 3.43 | 0.41 | 1.29 | 0.65 |
| 31.40 | 0.48 | 53.26 | 0.25 | 0.89 | 0.68 | 0.48 | 0.90 |
| 0.06 | 1.00 | 2.80 | 0.80 | 48.75 | 0.59 | 0.14 | 0.90 |
| 0.06 | 0.81 | 93.87 | 0.23 | 0.56 | 0.95 | 0.12 | 0.92 |
| 72.36 | 0.12 | 0.52 | 0.94 | 2.82 | 0.90 | 0.41 | 0.71 |
| 1.20 | 0.97 | 2.89 | 0.84 | 0.04 | 1.00 | 0.23 | 1.00 |
| 0.10 | 0.57 | 51.99 | 0.32 | 8.85 | 0.50 | 5.81 | 0.86 |
| 0.08 | 0.86 | 80.96 | 0.40 | 39.83 | 0.71 | 6.13 | 0.68 |
| 0.04 | 0.79 | 21.66 | 0.22 | 1.23 | 0.73 | 0.17 | 0.54 |
| 0.04 | 1.00 | 3.55 | 0.56 | 2.22 | 0.88 | 0.04 | 1.00 |
| 0.04 | 0.79 | 38.77 | 0.42 | 12.06 | 0.27 | 0.58 | 0.93 |
| 0.06 | 0.52 | 0.96 | 0.90 | 0.48 | 1.00 | 1.79 | 0.75 |
| 0.14 | 0.47 | 0.69 | 0.78 | 16.15 | 0.53 | 0.12 | 1.00 |
| 0.08 | 1.00 | 5.81 | 0.83 | 0.33 | 1.00 | 6.00 | 0.28 |
| 0.06 | 1.00 | 0.08 | 0.70 | 2.28 | 0.83 | 0.98 | 0.75 |
| 0.10 | 0.67 | 0.66 | 0.64 | 0.46 | 0.84 | 0.06 | 1.00 |
| 0.04 | 1.00 | 66.42 | 0.50 | 45.58 | 0.44 | 0.31 | 0.96 |
| 0.04 | 0.79 | 2.14 | 0.81 | 0.06 | 1.00 | 1.02 | 0.90 |
| 0.04 | 1.00 | 3.47 | 0.92 | 2.64 | 0.67 | 0.29 | 0.98 |
| 0.08 | 0.86 | 2.26 | 0.88 | 0.06 | 0.97 | 0.08 | 0.86 |
| 0.04 | 1.00 | 19.79 | 0.67 | 218.19 | 0.28 | 0.23 | 0.85 |
| 0.04 | 1.00 | 85.78 | 0.74 | 5.17 | 0.82 | 0.04 | 1.00 |
| 0.64 | 0.36 | 1.29 | 0.90 | 0.06 | 1.00 | 0.17 | 1.00 |
| 0.08 | 0.86 | 0.04 | 1.00 | 1.68 | 0.92 | 8.10 | 0.70 |
| 0.10 | 0.67 | 1.37 | 0.77 | 5.69 | 0.34 | 0.62 | 0.76 |
| 0.08 | 0.86 | 32.21 | 0.50 | 2.53 | 0.92 | 62.31 | 0.28 |
| 0.04 | 1.00 | 1.41 | 0.78 | 130.03 | 0.47 | 71.68 | 0.23 |
| 0.12 | 0.69 | 3.53 | 0.45 | 0.39 | 0.96 | 0.79 | 0.93 |
| 0.04 | 0.79 | 170.52 | 0.25 | 3.16 | 0.93 | 0.06 | 1.00 |
| 0.17 | 0.48 | 0.39 | 0.96 | 10.11 | 0.45 | 0.04 | 0.79 |
| 0.08 | 1.00 | 8.76 | 0.36 | 0.04 | 1.00 | 1.93 | 0.87 |
| 0.08 | 0.86 | 15.39 | 0.68 | 10.19 | 0.69 | 1.31 | 0.91 |
| 0.10 | 0.57 | 0.50 | 0.93 | 5.17 | 0.62 | 5.44 | 0.76 |
| 0.04 | 1.00 | 0.04 | 0.79 | 0.06 | 1.00 | 0.37 | 0.75 |
| 0.06 | 1.00 | 3.16 | 0.81 | 20.47 | 0.34 | 27.18 | 0.56 |
| 0.04 | 1.00 | 3.03 | 0.49 | 27.85 | 0.65 | 0.04 | 1.00 |
| 0.04 | 1.00 | 0.95 | 0.94 | 0.95 | 0.60 | 0.10 | 1.00 |
| 0.15 | 0.64 | 17.69 | 0.81 | 1.95 | 0.73 | 39.43 | 0.19 |
| 0.04 | 1.00 | 0.06 | 1.00 | 0.81 | 0.85 | 0.21 | 1.00 |
| 0.06 | 1.00 | 113.85 | 0.19 | 3.47 | 0.70 | 0.62 | 0.80 |
| 0.14 | 1.00 | 5.09 | 0.78 | 25.89 | 0.56 | 0.44 | 0.90 |
| 0.29 | 0.80 | 0.31 | 1.00 | 8.28 | 0.76 | 0.08 | 0.61 |
| 0.04 | 1.00 | 4.01 | 0.78 | 65.61 | 0.58 | 0.06 | 0.81 |
| 0.04 | 1.00 | 0.15 | 0.71 | 0.06 | 1.00 | 0.31 | 0.86 |
| 0.04 | 0.79 | 151.12 | 0.10 | 16.49 | 0.41 | 1.81 | 0.52 |
| 0.08 | 0.70 | 1.91 | 0.89 | 35.03 | 0.64 | 0.23 | 1.00 |
| 0.35 | 0.26 | 243.06 | 0.16 | 26.37 | 0.45 | 0.21 | 1.00 |
| 0.14 | 0.80 | 17.79 | 0.72 | 1.29 | 0.93 | 0.10 | 0.64 |
| 0.06 | 1.00 | 18.42 | 0.74 | 0.27 | 1.00 | 0.04 | 1.00 |
| 0.06 | 1.00 | 0.19 | 0.67 | 2.57 | 0.85 | 0.17 | 1.00 |
| 0.04 | 1.00 | 0.06 | 1.00 | 43.48 | 0.62 | 0.29 | 1.00 |
| 0.06 | 1.00 | 128.38 | 0.33 | 1.20 | 0.73 | 1.12 | 0.73 |
| 0.04 | 1.00 | 14.00 | 0.76 | 0.54 | 0.87 | 0.37 | 1.00 |
| 0.06 | 0.81 | 0.42 | 0.77 | 1.20 | 0.95 | 0.31 | 0.86 |
| 0.14 | 0.90 | 5.84 | 0.79 | 15.68 | 0.70 | 0.56 | 0.95 |
| 0.04 | 1.00 | 0.17 | 0.80 | 2.62 | 0.92 | 0.08 | 0.86 |
| 0.06 | 0.52 | 6.33 | 0.44 | 10.73 | 0.56 | 0.15 | 0.91 |
| 11.84 | 0.72 | 2.62 | 0.79 | 0.64 | 0.89 | 0.04 | 1.00 |
| 0.06 | 1.00 | 0.77 | 0.91 | 0.48 | 0.95 | 0.19 | 0.98 |
| 0.08 | 1.00 | 0.23 | 0.97 | 148.96 | 0.52 | 0.19 | 0.89 |
| 0.12 | 0.69 | 0.31 | 0.83 | 5.56 | 0.81 | 0.85 | 0.95 |
| 0.04 | 1.00 | 1.77 | 0.90 | 18.90 | 0.71 | 1.08 | 0.57 |
| 0.12 | 1.00 | 0.75 | 0.79 | 26.43 | 0.79 | 3.14 | 0.37 |
| 0.14 | 0.80 | 0.14 | 0.94 | 10.80 | 0.62 | 0.15 | 0.91 |
| 0.10 | 1.00 | 47.36 | 0.37 | 0.98 | 0.68 | 0.10 | 0.57 |
| 0.04 | 1.00 | 6.13 | 0.83 | 0.19 | 0.81 | 0.06 | 0.81 |
| 0.15 | 0.91 | 0.48 | 0.71 | 1.27 | 0.80 | 74.15 | 0.48 |
| 0.06 | 1.00 | 2.20 | 0.83 | 4.42 | 0.70 | 0.08 | 1.00 |
| 0.04 | 1.00 | 16.18 | 0.62 | 46.91 | 0.73 | 0.33 | 1.00 |
| 0.52 | 0.58 | 41.28 | 0.81 | 0.87 | 0.69 | 0.06 | 1.00 |
| 0.15 | 0.91 | 0.75 | 0.84 | 0.56 | 0.98 | 0.25 | 0.92 |
| 0.14 | 0.65 | 101.62 | 0.23 | 1.70 | 0.95 | 0.19 | 0.98 |
| 0.12 | 0.81 | 0.35 | 0.51 | 29.75 | 0.76 | 0.25 | 1.00 |
| 0.17 | 1.00 | 0.08 | 1.00 | 0.12 | 0.92 | 2.82 | 0.72 |
| 0.04 | 0.79 | 60.61 | 0.40 | 3.20 | 0.81 | 2.51 | 0.59 |
| 0.04 | 0.79 | 42.05 | 0.71 | 32.20 | 0.75 | 4.07 | 0.83 |
| 0.10 | 0.87 | 13.93 | 0.82 | 13.10 | 0.78 | 1.95 | 0.78 |
| 0.04 | 1.00 | 58.29 | 0.62 | 0.04 | 0.79 | 7.81 | 0.75 |
| 0.04 | 0.79 | 98.03 | 0.26 | 1.58 | 0.93 | 0.06 | 0.64 |
| 0.04 | 0.79 | 61.27 | 0.42 | 47.92 | 0.48 | 0.04 | 1.00 |
| 0.08 | 1.00 | 4.82 | 0.73 | 21.78 | 0.73 | 4.21 | 0.47 |
| 0.08 | 0.61 | 40.53 | 0.77 | 0.50 | 0.85 | 3.20 | 0.44 |
| 1.02 | 0.66 | 23.86 | 0.86 | 3.43 | 0.83 | 0.31 | 0.96 |
| 0.04 | 1.00 | 123.40 | 0.24 | 14.51 | 0.78 | 0.19 | 1.00 |
| 0.04 | 0.79 | 0.06 | 1.00 | 1.50 | 0.64 | 0.04 | 1.00 |
| 0.08 | 0.86 | 41.45 | 0.56 | 23.48 | 0.36 | 1.81 | 0.54 |
| 0.42 | 0.99 | 1.56 | 0.87 | 5.69 | 0.56 | 0.50 | 0.93 |
| 13.12 | 0.27 | 47.42 | 0.37 | 10.86 | 0.69 | 0.25 | 0.82 |
| 0.23 | 0.50 | 52.08 | 0.27 | 0.12 | 0.92 | 2.06 | 0.66 |
| 0.21 | 0.72 | 11.67 | 0.41 | 6.27 | 0.35 | 0.91 | 0.90 |
| 0.04 | 1.00 | 3.26 | 0.93 | 2.49 | 0.86 | 0.27 | 1.00 |
| 0.10 | 1.00 | 0.04 | 1.00 | 32.99 | 0.22 | 2.28 | 0.86 |
| 0.06 | 1.00 | 16.69 | 0.75 | 1.50 | 0.71 | 0.81 | 0.95 |
| 0.04 | 1.00 | 23.80 | 0.74 | 0.48 | 0.71 | 4.92 | 0.50 |
| 0.06 | 0.64 | 1.76 | 0.84 | 3.86 | 0.57 | 0.39 | 0.99 |
| 0.06 | 0.97 | 0.06 | 1.00 | 4.24 | 0.73 | 0.27 | 0.70 |
| 0.06 | 1.00 | 76.93 | 0.21 | 0.46 | 0.74 | 0.42 | 0.99 |
| 0.41 | 1.00 | 0.91 | 0.95 | 5.79 | 0.85 | 1.66 | 0.80 |
| 0.06 | 0.81 | 11.25 | 0.91 | 4.67 | 0.78 | 0.95 | 0.39 |
| 60.57 | 0.46 | 7.10 | 0.79 | 64.39 | 0.53 | 0.08 | 1.00 |
| 2.95 | 0.28 | 0.06 | 1.00 | 2.22 | 0.93 | 0.15 | 1.00 |
| 0.04 | 0.79 | 16.94 | 0.39 | 60.51 | 0.39 | 0.04 | 1.00 |
| 0.58 | 1.00 | 50.75 | 0.50 | 9.74 | 0.90 | 0.62 | 0.94 |
| 0.14 | 0.94 | 14.93 | 0.84 | 5.79 | 0.73 | 8.14 | 0.41 |
| 16.45 | 0.52 | 30.81 | 0.81 | 0.04 | 1.00 | 0.06 | 1.00 |
| 0.04 | 1.00 | 30.86 | 0.67 | 25.52 | 0.45 | 3.36 | 0.74 |
| 0.10 | 0.57 | 11.54 | 0.73 | 1.91 | 0.89 | 1.08 | 0.91 |
| 0.04 | 1.00 | 43.29 | 0.69 | 1.06 | 0.61 | 0.15 | 1.00 |
| 0.08 | 1.00 | 4.30 | 0.77 | 1.10 | 0.94 | 3.43 | 0.55 |
| 0.04 | 1.00 | 0.21 | 0.98 | 27.93 | 0.52 | 0.89 | 0.93 |
| 0.71 | 1.00 | 13.79 | 0.59 | 60.22 | 0.57 | 3.07 | 0.69 |
| 0.06 | 1.00 | 4.19 | 0.77 | 1.20 | 0.78 | 0.06 | 1.00 |
| 0.04 | 0.79 | 5.13 | 0.86 | 2.62 | 0.73 | 0.08 | 0.86 |
| 0.04 | 1.00 | 1.72 | 0.86 | 0.04 | 0.79 | 6.94 | 0.53 |
| 0.12 | 0.62 | 3.18 | 0.88 | 11.69 | 0.70 | 0.10 | 0.87 |
| 0.06 | 0.81 | 5.65 | 0.56 | 3.74 | 0.85 | 6.96 | 0.75 |
| 0.04 | 0.79 | 34.92 | 0.24 | 0.04 | 1.00 | 2.31 | 0.85 |
| 0.04 | 1.00 | 12.31 | 0.41 | 1.50 | 0.67 | 1.45 | 0.81 |
| 0.04 | 1.00 | 8.68 | 0.83 | 0.17 | 1.00 | 0.27 | 0.88 |
| 0.08 | 0.70 | 20.99 | 0.23 | 1.16 | 0.80 | 6.17 | 0.46 |
| 0.04 | 0.79 | 1.22 | 0.86 | 12.60 | 0.87 | 0.10 | 1.00 |
| 0.04 | 1.00 | 0.69 | 0.97 | 11.71 | 0.71 | 0.04 | 1.00 |
| 0.04 | 1.00 | 5.88 | 0.74 | 34.55 | 0.78 | 0.06 | 0.64 |
| 0.54 | 0.23 | 4.17 | 0.76 | 3.53 | 0.89 | 0.04 | 0.79 |
| 0.04 | 1.00 | 20.85 | 0.52 | 0.17 | 1.00 | 4.76 | 0.51 |
| 0.08 | 0.86 | 0.27 | 0.91 | 33.49 | 0.79 | 0.64 | 1.00 |
| 0.46 | 0.94 | 11.86 | 0.82 | 0.89 | 0.89 | 1.93 | 0.81 |
| 152.55 | 0.54 | 1.41 | 0.91 | 1.58 | 0.86 | 1.37 | 0.88 |
| 0.21 | 1.00 | 11.13 | 0.67 | 0.50 | 0.93 | 0.19 | 1.00 |
| 25.39 | 0.34 | 44.35 | 0.47 | 18.46 | 0.74 | 0.17 | 0.45 |
| 0.08 | 1.00 | 5.52 | 0.57 | 2.12 | 0.74 | 0.10 | 1.00 |
| 0.04 | 0.79 | 0.04 | 1.00 | 0.19 | 1.00 | 0.14 | 1.00 |
| 0.15 | 1.00 | 31.87 | 0.80 | 0.56 | 0.90 | 0.08 | 0.86 |
| 0.06 | 0.52 | 24.09 | 0.64 | 0.62 | 0.85 | 0.39 | 0.72 |
| 1.10 | 0.97 | 4.84 | 0.91 | 1.76 | 0.54 | 0.71 | 0.52 |
| 0.06 | 0.81 | 54.55 | 0.38 | 0.52 | 0.91 | 2.87 | 0.91 |
| 0.04 | 1.00 | 0.52 | 0.75 | 233.01 | 0.29 | 0.44 | 0.71 |
| 0.06 | 1.00 | 21.55 | 0.67 | 54.92 | 0.69 | 0.21 | 0.89 |
| 0.06 | 1.00 | 39.04 | 0.85 | 3.34 | 0.70 | 0.15 | 0.91 |
| 0.04 | 0.79 | 1.18 | 0.57 | 39.70 | 0.48 | 0.12 | 1.00 |
| 68.81 | 0.33 | 30.77 | 0.33 | 3.55 | 0.85 | 0.44 | 0.88 |
| 0.75 | 0.69 | 16.78 | 0.43 | 0.41 | 1.00 | 1.45 | 0.56 |
| 0.04 | 0.79 | 449.65 | 0.08 | 1.29 | 0.95 | 1.56 | 0.63 |
| 0.04 | 1.00 | 8.24 | 0.84 | 1.16 | 0.94 | 3.61 | 0.55 |
| 0.04 | 0.79 | 42.77 | 0.45 | 32.91 | 0.76 | 0.17 | 1.00 |
| 106.85 | 0.64 | 4.32 | 0.78 | 0.73 | 0.97 | 0.15 | 1.00 |
| 22.57 | 0.42 | 24.05 | 0.65 | 2.51 | 0.82 | 0.44 | 0.90 |
| 0.08 | 1.00 | 93.34 | 0.42 | 4.57 | 0.35 | 0.08 | 0.86 |
| 0.04 | 0.79 | 0.17 | 0.88 | 0.93 | 0.92 | 1.22 | 0.89 |
| 0.08 | 1.00 | 0.54 | 0.87 | 0.41 | 1.00 | 3.78 | 0.59 |
| 0.14 | 0.72 | 1.10 | 0.94 | 2.47 | 0.95 | 35.71 | 0.19 |
| 2.55 | 0.85 | 0.08 | 1.00 | 0.19 | 0.98 | 1.10 | 0.84 |
| 17.38 | 0.78 | 72.55 | 0.46 | 5.63 | 0.80 | 4.09 | 0.89 |
| 0.04 | 0.79 | 5.54 | 0.80 | 25.54 | 0.61 | 0.35 | 1.00 |
| 63.23 | 0.38 | 0.87 | 0.83 | 32.64 | 0.59 | 0.04 | 1.00 |
| 0.12 | 0.92 | 117.19 | 0.10 | 40.47 | 0.64 | 0.33 | 0.76 |
| 0.04 | 1.00 | 33.22 | 0.65 | 0.17 | 0.88 | 0.19 | 1.00 |
| 7.48 | 0.36 | 4.26 | 0.69 | 0.12 | 0.81 | 0.41 | 0.94 |
| 0.15 | 1.00 | 0.10 | 1.00 | 282.39 | 0.58 | 0.98 | 0.72 |
| 52.45 | 0.54 | 6.50 | 0.39 | 9.82 | 0.78 | 0.77 | 0.59 |
| 25.66 | 0.87 | 10.80 | 0.63 | 4.69 | 0.86 | 2.82 | 0.88 |
| 0.35 | 0.45 | 1.31 | 0.91 | 5.65 | 0.80 | 0.29 | 0.87 |
| 0.04 | 1.00 | 4.48 | 0.87 | 2.16 | 0.74 | 6.54 | 0.44 |
| 0.93 | 0.31 | 18.02 | 0.52 | 12.29 | 0.73 | 26.50 | 0.29 |
| 0.31 | 0.74 | 3.26 | 0.75 | 5.40 | 0.87 | 0.15 | 1.00 |
| 18.09 | 0.84 | 8.20 | 0.73 | 57.08 | 0.32 | 14.22 | 0.73 |
| 47.13 | 0.57 | 4.80 | 0.80 | 0.33 | 1.00 | 44.71 | 0.26 |
| 0.14 | 0.90 | 2.80 | 0.65 | 18.67 | 0.69 | 0.04 | 1.00 |
| 0.14 | 1.00 | 0.58 | 0.93 | 1.66 | 0.84 | 5.83 | 0.51 |
| 72.80 | 0.34 | 3.09 | 0.92 | 10.65 | 0.28 | 0.08 | 1.00 |
| 0.04 | 1.00 | 0.81 | 0.89 | 31.13 | 0.77 | 0.42 | 0.86 |
| 1.52 | 0.91 | 2.78 | 0.76 | 109.09 | 0.15 | 132.00 | 0.19 |
| 102.26 | 0.47 | 0.14 | 1.00 | 0.39 | 0.82 | 2.20 | 0.81 |
| 1.60 | 0.74 | 9.22 | 0.59 | 1.31 | 0.45 | 0.60 | 0.55 |
| 0.93 | 0.83 | 1.25 | 0.60 | 0.50 | 0.76 | 1.00 | 0.96 |
| 77.35 | 0.36 | 18.69 | 0.80 | 42.59 | 0.80 | 0.27 | 1.00 |
| 117.11 | 0.65 | 75.19 | 0.48 | 0.44 | 0.96 | 0.06 | 1.00 |
| 2.31 | 0.37 | 10.49 | 0.79 | 0.52 | 0.97 | 0.04 | 1.00 |
| 2.35 | 0.56 | 2.53 | 0.78 | 52.70 | 0.60 | 0.04 | 1.00 |
| 12.75 | 0.75 | 29.88 | 0.70 | 12.69 | 0.74 | 8.41 | 0.76 |
| 17.46 | 0.50 | 42.90 | 0.17 | 12.46 | 0.54 | 5.11 | 0.83 |
| 19.39 | 0.78 | 94.83 | 0.25 | 0.39 | 0.96 | 1.66 | 0.82 |
| 237.67 | 0.29 | 12.67 | 0.71 | 28.74 | 0.78 | 0.73 | 0.80 |
| 0.69 | 0.51 | 20.00 | 0.73 | 0.12 | 0.81 | 0.98 | 0.96 |
| 199.83 | 0.69 | 0.06 | 1.00 | 10.76 | 0.87 | 0.15 | 0.54 |
| 0.54 | 0.92 | 55.36 | 0.34 | 14.47 | 0.76 | 0.19 | 1.00 |
| 103.41 | 0.39 | 0.10 | 1.00 | 8.31 | 0.70 | 0.50 | 0.79 |
| 0.04 | 1.00 | 0.25 | 0.60 | 1.52 | 0.90 | 2.01 | 0.82 |
| 324.83 | 0.66 | 4.40 | 0.58 | 0.77 | 0.89 | 2.80 | 0.85 |
| 32.16 | 0.53 | 1.14 | 0.44 | 1.83 | 0.59 | 0.19 | 1.00 |
| 60.80 | 0.59 | 0.10 | 0.57 | 28.59 | 0.48 | 0.42 | 0.92 |
| 1.68 | 0.89 | 0.44 | 0.96 | 16.86 | 0.41 | 0.46 | 0.51 |
| 53.20 | 0.27 | 3.61 | 0.72 | 426.45 | 0.47 | 0.31 | 0.80 |
| 13.23 | 0.15 | 0.46 | 0.94 | 30.65 | 0.50 | 1.18 | 0.83 |
| 81.10 | 0.22 | 163.93 | 0.25 | 14.37 | 0.47 | 11.38 | 0.49 |
| 2.08 | 0.85 | 0.64 | 0.73 | 29.94 | 0.32 | 1.74 | 0.32 |
| 8.02 | 0.86 | 6.73 | 0.67 | 1.72 | 0.82 | 0.12 | 0.69 |
| 39.26 | 0.48 | 22.26 | 0.84 | 2.16 | 0.73 | 3.63 | 0.74 |
| 1.06 | 0.91 | 16.24 | 0.83 | 67.11 | 0.50 | 2.91 | 0.77 |
| 0.25 | 0.70 | 0.46 | 0.98 | 20.66 | 0.69 | 0.19 | 0.71 |
| 0.15 | 1.00 | 0.08 | 1.00 | 4.88 | 0.65 | 2.22 | 0.29 |
| 1.00 | 0.88 | 1.27 | 0.90 | 0.37 | 0.80 | 0.71 | 0.80 |
| 30.42 | 0.52 | 5.17 | 0.82 | 4.46 | 0.68 | 0.14 | 1.00 |
| 0.23 | 1.00 | 0.75 | 0.86 | 18.00 | 0.57 | 36.05 | 0.58 |
| 340.97 | 0.08 | 4.38 | 0.83 | 15.82 | 0.70 | 0.06 | 0.81 |
| 0.17 | 1.00 | 20.04 | 0.58 | 0.29 | 0.98 | 1.83 | 0.95 |
| 849.09 | 0.09 | 113.93 | 0.31 | 2.97 | 0.94 | 0.41 | 1.00 |
| 55.23 | 0.80 | 88.00 | 0.33 | 16.72 | 0.45 | 0.64 | 0.94 |
| 54.94 | 0.27 | 17.28 | 0.29 | 0.27 | 0.99 | 6.71 | 0.82 |
| 0.39 | 0.67 | 17.01 | 0.63 | 0.64 | 0.97 | 0.14 | 0.94 |
| 57.60 | 0.34 | 9.03 | 0.43 | 0.46 | 0.94 | 0.12 | 0.92 |
| 0.10 | 1.00 | 3.92 | 0.69 | 67.05 | 0.26 | 0.06 | 1.00 |
| 88.46 | 0.28 | 600.25 | 0.07 | 0.17 | 0.92 | 0.06 | 1.00 |
| 0.17 | 1.00 | 154.26 | 0.24 | 5.07 | 0.56 | 5.90 | 0.31 |
| 1.79 | 0.96 | 2.10 | 0.91 | 1.10 | 0.87 | 3.55 | 0.91 |
| 0.06 | 1.00 | 0.83 | 0.71 | 11.25 | 0.63 | 0.04 | 1.00 |
| 84.36 | 0.31 | 7.91 | 0.80 | 4.71 | 0.57 | 8.20 | 0.50 |
| 0.27 | 0.50 | 2.39 | 0.88 | 0.39 | 0.90 | 0.41 | 0.94 |
| 62.06 | 0.40 | 16.84 | 0.26 | 0.04 | 1.00 | 0.50 | 0.81 |
| 123.24 | 0.38 | 0.37 | 0.80 | 1.56 | 0.82 | 25.75 | 0.72 |
| 0.14 | 1.00 | 10.46 | 0.67 | 0.21 | 1.00 | 1.41 | 0.96 |
| 162.77 | 0.40 | 2.87 | 0.70 | 2.84 | 0.46 | 0.08 | 0.86 |
| 48.26 | 0.48 | 5.32 | 0.90 | 3.38 | 0.84 | 0.21 | 0.98 |
| 0.35 | 0.89 | 15.12 | 0.83 | 2.60 | 0.93 | 0.04 | 0.79 |
| 0.56 | 0.98 | 11.59 | 0.53 | 27.99 | 0.67 | 4.38 | 0.40 |
| 0.10 | 1.00 | 2.60 | 0.80 | 14.29 | 0.50 | 4.32 | 0.66 |
| 0.35 | 1.00 | 5.81 | 0.94 | 8.35 | 0.84 | 1.50 | 0.72 |
| 28.70 | 0.39 | 6.44 | 0.78 | 0.77 | 0.69 | 0.04 | 1.00 |
| 0.52 | 1.00 | 1.22 | 0.77 | 73.21 | 0.19 | 15.03 | 0.57 |
| 0.39 | 0.84 | 48.21 | 0.13 | 2.49 | 0.46 | 0.37 | 0.80 |
| 0.21 | 1.00 | 0.19 | 1.00 | 29.82 | 0.20 | 0.12 | 0.77 |
| 0.48 | 0.98 | 65.90 | 0.37 | 5.27 | 0.57 | 1.02 | 0.96 |
| 0.83 | 0.85 | 95.49 | 0.22 | 1.79 | 0.96 | 0.14 | 1.00 |
| 0.52 | 1.00 | 0.54 | 0.87 | 6.29 | 0.76 | 1.68 | 0.67 |
| 29.44 | 0.62 | 0.56 | 1.00 | 7.97 | 0.64 | 3.95 | 0.85 |
| 73.17 | 0.41 | 4.22 | 0.69 | 15.63 | 0.87 | 1.20 | 0.79 |
| 1.10 | 0.66 | 229.22 | 0.33 | 1.89 | 0.92 | 0.08 | 0.70 |
| 0.29 | 1.00 | 0.91 | 0.92 | 27.57 | 0.50 | 0.15 | 1.00 |
| 25.39 | 0.43 | 34.05 | 0.55 | 2.49 | 0.73 | 9.76 | 0.68 |
| 14.10 | 0.64 | 0.06 | 1.00 | 0.23 | 1.00 | 2.06 | 0.89 |
| 0.69 | 0.78 | 71.53 | 0.43 | 71.41 | 0.28 | 0.31 | 0.96 |
| 0.96 | 0.78 | 14.80 | 0.71 | 6.31 | 0.55 | 1.12 | 0.50 |
| 62.27 | 0.35 | 0.19 | 1.00 | 0.08 | 0.64 | 6.91 | 0.58 |
| 166.05 | 0.20 | 21.06 | 0.42 | 26.04 | 0.76 | 23.98 | 0.28 |
| 0.31 | 1.00 | 15.84 | 0.90 | 0.29 | 0.90 | 2.58 | 0.87 |
| 0.23 | 1.00 | 4.84 | 0.75 | 66.80 | 0.14 | 0.15 | 1.00 |
| 0.62 | 0.66 | 0.85 | 0.89 | 0.21 | 1.00 | 2.08 | 0.92 |
| 0.42 | 0.86 | 220.87 | 0.17 | 0.29 | 0.49 | 0.58 | 1.00 |
| 67.19 | 0.21 | 0.14 | 0.90 | 13.46 | 0.80 | 0.15 | 0.91 |
| 15.53 | 0.63 | 13.95 | 0.78 | 9.51 | 0.58 | 0.08 | 1.00 |
| 0.19 | 0.98 | 0.14 | 1.00 | 0.54 | 0.46 | 3.84 | 0.81 |
| 16.67 | 0.75 | 12.33 | 0.49 | 0.64 | 0.89 | 0.42 | 0.92 |
| 0.54 | 0.87 | 5.90 | 0.41 | 5.05 | 0.55 | 2.49 | 0.60 |
| 1.56 | 0.89 | 47.32 | 0.24 | 1.74 | 0.75 | 0.15 | 1.00 |
| 18.38 | 0.90 | 1.00 | 0.56 | 0.50 | 0.81 | 2.91 | 0.90 |
| 0.19 | 1.00 | 0.79 | 0.87 | 3.11 | 0.81 | 23.88 | 0.27 |
| 11.59 | 0.79 | 2.91 | 0.85 | 0.14 | 0.80 | 0.23 | 0.85 |
| 0.19 | 0.81 | 2.14 | 0.92 | 0.23 | 0.97 | 0.37 | 0.64 |
| 0.14 | 1.00 | 1.85 | 0.86 | 10.76 | 0.39 | 5.00 | 0.82 |
| 107.54 | 0.42 | 14.87 | 0.38 | 52.66 | 0.29 | 0.08 | 0.46 |
| 0.29 | 0.98 | 24.00 | 0.43 | 0.04 | 1.00 | 0.12 | 1.00 |
| 152.31 | 0.17 | 29.63 | 0.47 | 0.15 | 1.00 | 0.17 | 1.00 |
| 2.87 | 0.95 | 211.92 | 0.18 | 1.99 | 0.81 | 0.31 | 0.86 |
| 0.60 | 0.96 | 102.41 | 0.37 | 12.25 | 0.84 | 0.14 | 0.94 |
| 66.01 | 0.43 | 50.42 | 0.46 | 12.09 | 0.49 | 0.08 | 1.00 |
| 45.60 | 0.47 | 2.14 | 0.81 | 0.08 | 1.00 | 5.38 | 0.45 |
| 41.30 | 0.64 | 5.90 | 0.44 | 2.80 | 0.85 | 1.50 | 0.95 |
| 0.19 | 1.00 | 37.35 | 0.70 | 24.85 | 0.61 | 0.06 | 1.00 |
| 0.10 | 1.00 | 1.95 | 0.85 | 1.25 | 0.72 | 3.28 | 0.35 |
| 0.10 | 0.87 | 0.96 | 1.00 | 204.82 | 0.23 | 1.31 | 0.79 |
| 23.57 | 0.76 | 17.80 | 0.89 | 0.48 | 0.98 | 0.37 | 0.94 |
| 0.04 | 0.79 | 7.18 | 0.78 | 1.22 | 0.84 | 3.59 | 0.75 |
| 317.50 | 0.14 | 1.37 | 0.88 | 88.77 | 0.51 | 0.08 | 1.00 |
| 17.77 | 0.42 | 0.44 | 1.00 | 4.80 | 0.91 | 4.53 | 0.89 |
| 102.03 | 0.23 | 6.06 | 0.57 | 1.12 | 0.82 | 6.12 | 0.84 |
| 0.85 | 0.89 | 1.43 | 0.91 | 11.42 | 0.79 | 3.28 | 0.40 |
| 80.32 | 0.60 | 0.15 | 1.00 | 24.48 | 0.50 | 70.45 | 0.69 |
| 97.59 | 0.16 | 0.17 | 0.83 | 0.98 | 0.90 | 0.21 | 1.00 |
| 0.19 | 0.98 | 3.88 | 0.91 | 31.29 | 0.54 | 0.27 | 0.59 |
| 15.82 | 0.24 | 0.21 | 0.85 | 0.08 | 1.00 | 1.00 | 0.57 |
| 19.35 | 0.59 | 8.20 | 0.79 | 35.92 | 0.73 | 2.45 | 0.89 |
| 297.96 | 0.18 | 0.35 | 0.87 | 3.99 | 0.88 | 0.15 | 1.00 |
| 14.91 | 0.33 | 518.04 | 0.10 | 0.06 | 1.00 | 0.04 | 1.00 |
| 22.26 | 0.81 | 2.33 | 0.57 | 0.54 | 1.00 | 22.16 | 0.37 |
| 344.66 | 0.20 | 26.41 | 0.54 | 67.21 | 0.32 | 1.68 | 0.85 |
| 0.12 | 1.00 | 1.58 | 0.97 | 0.91 | 0.86 | 1.62 | 0.39 |
| 0.46 | 0.86 | 0.58 | 0.68 | 12.73 | 0.34 | 0.19 | 1.00 |
| 22.22 | 0.63 | 2.31 | 0.80 | 24.25 | 0.33 | 0.54 | 1.00 |
| 0.15 | 0.82 | 2.30 | 0.93 | 64.62 | 0.31 | 1.08 | 0.66 |
| 12.37 | 0.47 | 3.11 | 0.60 | 2.20 | 0.81 | 0.04 | 1.00 |
| 0.14 | 1.00 | 19.06 | 0.78 | 0.10 | 1.00 | 0.14 | 0.94 |
| 0.12 | 1.00 | 3.55 | 0.74 | 0.10 | 1.00 | 0.68 | 0.71 |
| 0.71 | 1.00 | 4.98 | 0.70 | 0.10 | 0.67 | 0.08 | 1.00 |
| 1.66 | 0.64 | 150.79 | 0.24 | 95.14 | 0.36 | 7.56 | 0.78 |
| 0.08 | 0.86 | 80.23 | 0.14 | 5.23 | 0.76 | 0.19 | 0.89 |
| 166.05 | 0.17 | 11.59 | 0.84 | 0.19 | 0.98 | 0.04 | 1.00 |
| 0.60 | 0.74 | 14.41 | 0.69 | 15.08 | 0.43 | 10.57 | 0.63 |
| 11.38 | 0.53 | 23.15 | 0.42 | 1.14 | 0.87 | 0.39 | 0.66 |
| 0.17 | 1.00 | 1.00 | 0.57 | 10.67 | 0.61 | 85.20 | 0.25 |
| 0.37 | 0.85 | 180.17 | 0.12 | 3.68 | 0.54 | 25.95 | 0.36 |
| 0.48 | 0.64 | 16.13 | 0.81 | 0.10 | 1.00 | 0.12 | 1.00 |
| 0.10 | 1.00 | 9.16 | 0.56 | 2.26 | 0.69 | 1.08 | 0.82 |
| 0.60 | 0.84 | 25.75 | 0.51 | 2.78 | 0.80 | 0.69 | 1.00 |
| 0.27 | 0.99 | 0.66 | 0.85 | 0.62 | 0.58 | 0.17 | 0.88 |
| 0.27 | 0.75 | 0.04 | 1.00 | 2.95 | 0.57 | 0.14 | 0.50 |
| 131.60 | 0.37 | 1.54 | 0.90 | 134.55 | 0.11 | 0.91 | 0.95 |
| 0.06 | 1.00 | 3.34 | 0.69 | 3.14 | 0.73 | 0.10 | 1.00 |
| 0.69 | 0.92 | 1.62 | 0.79 | 6.60 | 0.88 | 0.25 | 1.00 |
| 0.06 | 1.00 | 17.94 | 0.77 | 12.21 | 0.63 | 1.47 | 0.91 |
| 4.65 | 0.46 | 0.14 | 0.90 | 1.37 | 0.82 | 0.10 | 0.87 |
| 2.30 | 0.79 | 16.24 | 0.47 | 16.09 | 0.41 | 0.41 | 1.00 |
| 119.46 | 0.55 | 24.44 | 0.49 | 1.83 | 0.80 | 0.68 | 0.95 |
| 0.06 | 1.00 | 39.62 | 0.70 | 5.65 | 0.40 | 28.13 | 0.35 |
| 0.14 | 1.00 | 6.35 | 0.76 | 5.30 | 0.87 | 0.39 | 0.67 |
| 57.48 | 0.29 | 13.58 | 0.58 | 0.73 | 0.86 | 3.72 | 0.45 |
| 1.77 | 0.75 | 68.71 | 0.21 | 0.35 | 1.00 | 0.27 | 1.00 |
| 40.14 | 0.50 | 1.16 | 0.94 | 2.31 | 0.74 | 4.94 | 0.72 |
| 34.57 | 0.50 | 11.09 | 0.65 | 0.15 | 1.00 | 2.14 | 0.53 |
| 0.50 | 1.00 | 0.87 | 0.73 | 10.67 | 0.36 | 0.46 | 0.94 |
| 193.27 | 0.23 | 0.04 | 1.00 | 18.06 | 0.53 | 0.06 | 1.00 |
| 0.23 | 1.00 | 24.90 | 0.68 | 0.42 | 0.56 | 0.25 | 1.00 |
| 0.06 | 1.00 | 2.33 | 0.73 | 56.85 | 0.26 | 0.50 | 0.96 |
| 0.25 | 1.00 | 0.62 | 0.99 | 6.40 | 0.68 | 3.09 | 0.83 |
| 64.78 | 0.20 | 1.93 | 0.91 | 6.60 | 0.93 | 0.19 | 0.89 |
| 3.40 | 0.68 | 1.12 | 0.82 | 4.09 | 0.83 | 5.94 | 0.56 |
| 0.87 | 0.73 | 1.70 | 0.38 | 82.50 | 0.37 | 0.27 | 0.99 |
| 0.12 | 1.00 | 17.05 | 0.79 | 0.08 | 1.00 | 0.15 | 1.00 |
| 4.63 | 0.86 | 85.17 | 0.30 | 0.12 | 0.92 | 3.03 | 0.87 |
| 1.00 | 0.92 | 255.34 | 0.24 | 2.70 | 0.62 | 0.95 | 0.51 |
| 0.56 | 0.95 | 9.39 | 0.62 | 12.50 | 0.43 | 0.83 | 0.93 |
| 66.53 | 0.22 | 1.08 | 0.73 | 7.29 | 0.47 | 0.31 | 1.00 |
| 0.83 | 0.91 | 11.03 | 0.73 | 0.42 | 1.00 | 22.05 | 0.37 |
| 0.39 | 1.00 | 2.01 | 0.88 | 0.06 | 1.00 | 1.04 | 0.95 |
| 0.15 | 1.00 | 208.33 | 0.67 | 36.21 | 0.40 | 3.43 | 0.88 |
| 0.37 | 1.00 | 0.87 | 0.28 | 50.56 | 0.38 | 0.12 | 1.00 |
| 6.21 | 0.82 | 4.71 | 0.67 | 0.08 | 0.86 | 1.00 | 0.90 |
| 1.23 | 0.87 | 63.95 | 0.49 | 5.88 | 0.75 | 0.29 | 0.80 |
| 0.19 | 0.98 | 1.95 | 0.89 | 1.39 | 0.71 | 0.60 | 0.79 |
| 68.38 | 0.42 | 22.15 | 0.60 | 0.42 | 0.58 | 8.93 | 0.76 |
| 133.66 | 0.26 | 6.31 | 0.38 | 60.98 | 0.38 | 0.64 | 1.00 |
| 0.33 | 0.98 | 1.14 | 0.96 | 1.95 | 0.54 | 0.08 | 1.00 |
| 1.50 | 0.89 | 8.91 | 0.89 | 2.51 | 0.69 | 0.52 | 0.97 |
| 57.91 | 0.20 | 15.41 | 0.71 | 1.68 | 0.69 | 0.42 | 0.79 |
| 3.72 | 0.60 | 0.95 | 0.88 | 0.62 | 0.80 | 23.59 | 0.37 |
| 0.14 | 0.94 | 55.65 | 0.40 | 70.14 | 0.24 | 0.04 | 1.00 |
| 40.63 | 0.86 | 0.19 | 0.48 | 0.62 | 0.63 | 33.78 | 0.57 |
| 78.26 | 0.24 | 112.52 | 0.29 | 2.12 | 0.86 | 0.29 | 0.69 |
| 0.29 | 1.00 | 0.29 | 0.65 | 1.31 | 1.00 | 0.95 | 0.64 |
| 20.41 | 0.83 | 15.10 | 0.26 | 23.46 | 0.74 | 0.12 | 0.81 |
| 169.62 | 0.32 | 0.08 | 1.00 | 0.21 | 1.00 | 1.83 | 0.86 |
| 36.90 | 0.39 | 104.84 | 0.37 | 1.12 | 0.82 | 0.06 | 0.81 |
| 1.97 | 0.80 | 0.08 | 0.74 | 63.04 | 0.11 | 1.04 | 0.97 |
| 0.06 | 1.00 | 27.04 | 0.45 | 4.19 | 0.77 | 1.81 | 0.47 |
| 22.28 | 0.32 | 0.21 | 0.72 | 45.52 | 0.83 | 1.58 | 0.65 |
| 85.92 | 0.30 | 12.40 | 0.69 | 8.97 | 0.80 | 0.23 | 1.00 |
| 158.82 | 0.18 | 1.77 | 0.79 | 5.75 | 0.74 | 0.54 | 0.73 |
| 140.14 | 0.36 | 31.40 | 0.33 | 1.27 | 0.90 | 0.35 | 0.96 |
| 0.04 | 1.00 | 2.68 | 0.85 | 3.90 | 0.56 | 0.25 | 0.75 |
| 0.06 | 0.97 | 0.81 | 1.00 | 85.28 | 0.32 | 2.72 | 0.61 |
| 37.56 | 0.77 | 0.41 | 0.94 | 0.66 | 0.92 | 0.64 | 0.97 |
| 0.12 | 1.00 | 0.04 | 1.00 | 61.88 | 0.35 | 28.14 | 0.68 |
| 0.83 | 0.95 | 14.54 | 0.61 | 9.16 | 0.71 | 3.03 | 0.72 |
| 187.81 | 0.19 | 15.76 | 0.64 | 0.31 | 0.93 | 0.89 | 0.97 |
| 119.14 | 0.21 | 3.18 | 0.91 | 6.19 | 0.44 | 0.33 | 0.91 |
| 66.20 | 0.24 | 14.20 | 0.75 | 2.89 | 0.82 | 1.52 | 0.93 |
| 1.31 | 0.89 | 0.85 | 0.82 | 0.58 | 0.55 | 0.48 | 0.95 |
| 4.82 | 0.86 | 7.58 | 0.76 | 1.22 | 0.87 | 0.06 | 0.64 |
| 10.73 | 0.60 | 0.35 | 1.00 | 0.10 | 0.87 | 0.08 | 1.00 |
| 37.33 | 0.51 | 0.41 | 0.94 | 0.23 | 0.85 | 21.01 | 0.53 |
| 0.98 | 0.92 | 0.60 | 0.81 | 1.81 | 0.77 | 41.40 | 0.24 |
| 83.66 | 0.25 | 1.62 | 0.22 | 23.13 | 0.74 | 0.35 | 0.81 |
| 31.33 | 0.66 | 0.19 | 0.55 | 0.27 | 0.75 | 1.85 | 0.93 |
| 0.21 | 0.98 | 0.04 | 1.00 | 140.95 | 0.26 | 0.33 | 1.00 |
| 89.54 | 0.47 |  |  | 16.53 | 0.83 | 0.48 | 0.82 |
